# Mechanical characteristics analysis of coal around borehole based on three-stage constitutive model and its numerical validation

**DOI:** 10.1038/s41598-025-98988-9

**Published:** 2025-05-06

**Authors:** XiuFeng Zhang, Yulin Lu, Haichen Yin, Xun Guo, Hao Wang, Yang Chen

**Affiliations:** 1 Coal Industry Management Department, Shandong Energy Group Co., Ltd, Jinan, 250014 China; 2 School of Civil Engineering, Institute of Disaster Prevention, Sanhe, 065201 China; 3 Hebei Technology Innovation Center for Multi-Hazard Resilience and Emergency Handling of Engineering Structures, Sanhe, 065201 China

**Keywords:** Analytical solution, Three-stage constitutive model, Elastic–plastic analysis, Coal around borehole, Numerical validation, Energy science and technology, Engineering

## Abstract

The stress and deformation characteristics of coal around borehole have an important impact on the accuracy of stress monitoring in deep mining engineering. This study aims to analyze the stress and deformation of coal around borehole under different stress conditions and the influences of various parameters on deformation distribution by applying an analytical method. Firstly, the linearization of complete stress–strain model of coal is curried out according to the field observation data, and the simplified constitutive model is divided into three stages, i.e., elastic, plastic and broken stage. Secondly, based on the elastic–plastic theory, an analytical solution of coal around borehole includes stress and deformation of the elastic, plastic softening, and broken region is deduced, and the effects of parameters such as lateral pressure coefficient, intermediate principal stress coefficient, vertical stress, borehole radius, cohesion, and internal friction angle on radii of different regions are discussed in detail. Thirdly, the analytical results show that only the lateral pressure coefficient affects the deformation shape of coal around borehole, and other parameters have almost no influence on the deformation shape; A large lateral pressure coefficient corresponds to a small radius of the broken and plastic softening region; The radii of broken and plastic softening regions increase significantly with an increase in vertical stress but decrease clearly with an increase in cohesion and internal friction angle; Similarly, the larger the borehole radius, the greater the deformation ranges; The radii of two regions are not monotonous with the intermediate principal stress coefficient, there is almost an inflection point for the intermediate principal stress coefficient corresponding to the minimum radii of two regions, and the value is about 0.7. Finally, a two-dimensional numerical solution of coal around borehole is also employed by using the finite element method, and the radial and tangential stress calculated by FEM are essentially consistent with the results of the analytical method, and the maximum error is approximately 10.56%. Numerical results prove the rationality of the analytical method proposed in this study.

## Introduction

With the development of deep mining engineering, the stress monitoring technique has become an important means of evaluation the safety and stability of coal. However, the deformation and damage of coal around borehole will significantly affect stress monitoring efficacy^1^ It is widely known that drilling destroys the balance of original stress, then promotes secondary distribution of stress surrounding coal and generates stress concentration region around the borehole^2,3^ Serious high stress of coal even creates huge mine hazards and results in the loss of a great deal of economic in subsequent mining work. Therefore, a study of the mechanical behavior and failure mechanism of deep coal under drilling disturbance is of great importance both in theory and application for assessing the safety and stability of coal.

Unit now, the elastic–plastic solution is a key to analyzing the deformation of coal around borehole, and this is similar to the stability problem of surrounding rock in roadway. Since the 1930s, large numbers of researchers have conducted many studies on the surrounding rock mechanics problems and proposed various calculated methods. Based on Mohr–Coulomb criterion, a plastic region range of surrounding rock in the circular tunnel under a uniform stress field with ideal elastic–plastic conditions was first reported by Fenner,^4^ and then some amended measures were proposed by Kastner^5^ because this calculation model was too simplified. Over the past three decades, many scholars, such as Sharan et al., Park et al., Jiang et al., Sharan et al., Chen et al., and Wang et al., also applied this approach combining different constitutive models and associated flow rule or non-associated flow rule to study the deformation distribution characteristic and displacement solution of surrounding rock^6–11^ The above research mainly focuses on the working conditions of a uniform stress field, which is a very conservative assumption and is inconsistent with the facts, limiting the result’s precision and application. A lot of observed data indicate that horizontal stress is not usually equal to vertical stress in deep rock, which means that the surrounding rock is subjected to a non-uniform stress field. To overcome the drawback of this model, Kirsch^12^ conducted elastic theory to study the deformation characteristics of surrounding rock in a circular tunnel under a non-uniform stress field, Kastner^5^ provided an approximate implicit solution for the plastic boundary of surrounding rock, Simanjuntak^13^ also given a series of the related solution includes distribution of stresses, strains, and deformations during tunnelling processes by applying finite element approach. Pan et al., Shen., Fan et al., Wang et al., and Xu et al., also successively developed a calculated method of the non-uniform stress field in an analysis of surrounding rock, which provided an elastic–plastic solution of deformation of rock mass, and verified the feasibility of this method through practical cases or numerical simulation in recent years^14–18^ It is worth noting that the above mentioned research objects are mainly surrounding rocks in the tunnel, its mechanical behaviors are still different from those coals around the borehole. Due to the effects of disturbance caused by drilling, the mechanical evolution and deformation failure characteristics of deep coal are very complex, which brings a representative problem in how to accurately monitor the stress and deformation of coal. However, academic circles have not thought highly of the research on this problem, related results are limited.

The plastic strain softening model of coal is another center of attention in the analysis of mechanical characteristics, and it has been investigated widely by numerical, analytical, and experimental approaches since the 1960s. Unlike the conventional elastic-perfectly plastic model, softening is a gradually weakening process once the stress strength exceeds peak value^19,20^ Diest^21^ was one of the earliest scholars who employed the softening model to study failure zones around deep-level mining excavations, and Brown et al^22^ also proposed an implicit solution of softening and broken regions by using non-linear peak and residual rock strength standard based on Hoek–Brown criterion. Along with the computational mechanic development and technological progress, by virtue of the finite element method, the softening model has been well studied in the elastoplastic analysis of surrounding rock. Zhang et al^23^ presented a numerical finite strain solution for a circular tunnel in Mohr–Coulomb and generalized Hoek–Brown criterion, and it was verified by another numerical analysis method. Deng et al^24^ introduced a variable critical plastic softening parameter to investigate the elastoplastic behavior of the circular tunnel excavation, while also proposing a numerical procedure to verify its rationality. Li et al^25^ carried out an elastic-strain softening model to analyze the failure, plastic, and elastic zone in surrounding rock, and discussed the stress distribution of three zones under the conditions of compaction and expansion. Although these models have achieves some achievement, there is some debate about the variation rule of cohesion and internal friction angle in plastic softening region, and is no unified explanation and exact conclusion yet. Some researchers such as González et al., Jing et al., Cui et al., and Xu et al. have suggested that the essence of strain-softening was that the internal friction angle remains unchanged while the cohesion decreases, thus the residual internal friction angle was not taken into consideration in plastic analysis^18,26–28^ However, contrary to the above view, many scholars argue that the internal friction angle is also regarded as a major softening parameter in addition to the attenuation of cohesion. Li et al^29^ pointed out that the internal friction angle and cohesion have the same variation trend with rock strength, which decreased with the decrease of rock strength in the softening stage, and a nonlinear post-peak stress–strain relationship of rock was built on this basis. Yang et al^30^ presented a damage weaken equation for post-peak deformation based on the residual parameters of the softening stage, and established a damage-softening constitutive model of rock, which was verified through experiments. Fan et al^16^ also applied a new constitutive model based on the strain softening relation that simultaneously considered the weakening of strength parameters to deduce the analytic solution of the radii and displacement in the post-peak softening region, and the results show that the radii and displacement all decreased with increasing residual internal friction angle. Therefore, how to describe quantitatively the softening parameters effects on stress and deformation of coal around borehole is a key part of the softening model, and this problem needs further discussion.

In a word, the non-uniform stress field and plastic softening model are two problems that are focused on the analysis of the stress and deformation of coal around borehole. Therefore, an analytical solution that includes stress and deformation of the broken, plastic softening, and elastic region is proposed to consider the influences of different stress conditions, plastic softening strength parameters, and intermediate principal stress on the mechanical characteristics of coal around borehole in this study. To test the accuracy of the analytical method, a numerical procedure based on Drucker-Prager strength criterion is executed to determine the stress field of coal around borehole. In the end, the results of two methods and the sensitivity of various parameters on the mechanical characteristics of coal around borehole are analyzed in detail. This present study will provide a foundation for further theoretical studies on the stress monitoring techniques.

## Basic principle and calculation method

### Mechanical model of coal around borehole

**Fig. 1:**
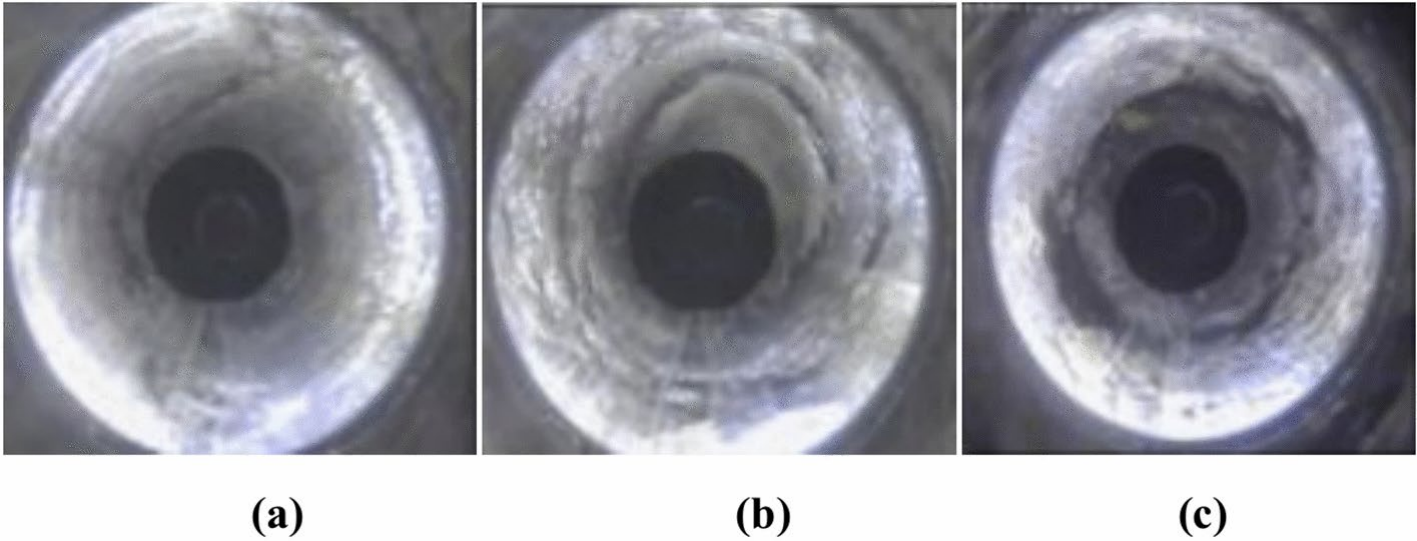
Deformation state of coal around borehole^31^. (**a**) intact state; (**b**) damage state; (**c**) broken state.

In the stress monitoring process, drilling usually causes stress release, which has changed the pattern of stress distribution and even destroyed the original stress state. Therefore, the coal stress field around borehole is very complex. Li^31^ found a clear deformation state in different depths of coal during the mining process by using the borehole observation instrument, and it was described as three states, i.e., intact, damaged, and broken state, as shown in Fig. 1. The broken coal remains in its original position and has not fallen into the borehole, which is different from the results researched by Lakirouhani^32–35^ This phenomenon is consisttent with the theory of continuum mechanics. According to the stress–strain relationship of coal, the intact, damaged, and broken state, which are correspond with the elastic, plastic, and residual deformation of coal, respectively. This provides a theoretical basis for simplifying the constitutive relationship of the three stages.

**Fig. 2:**
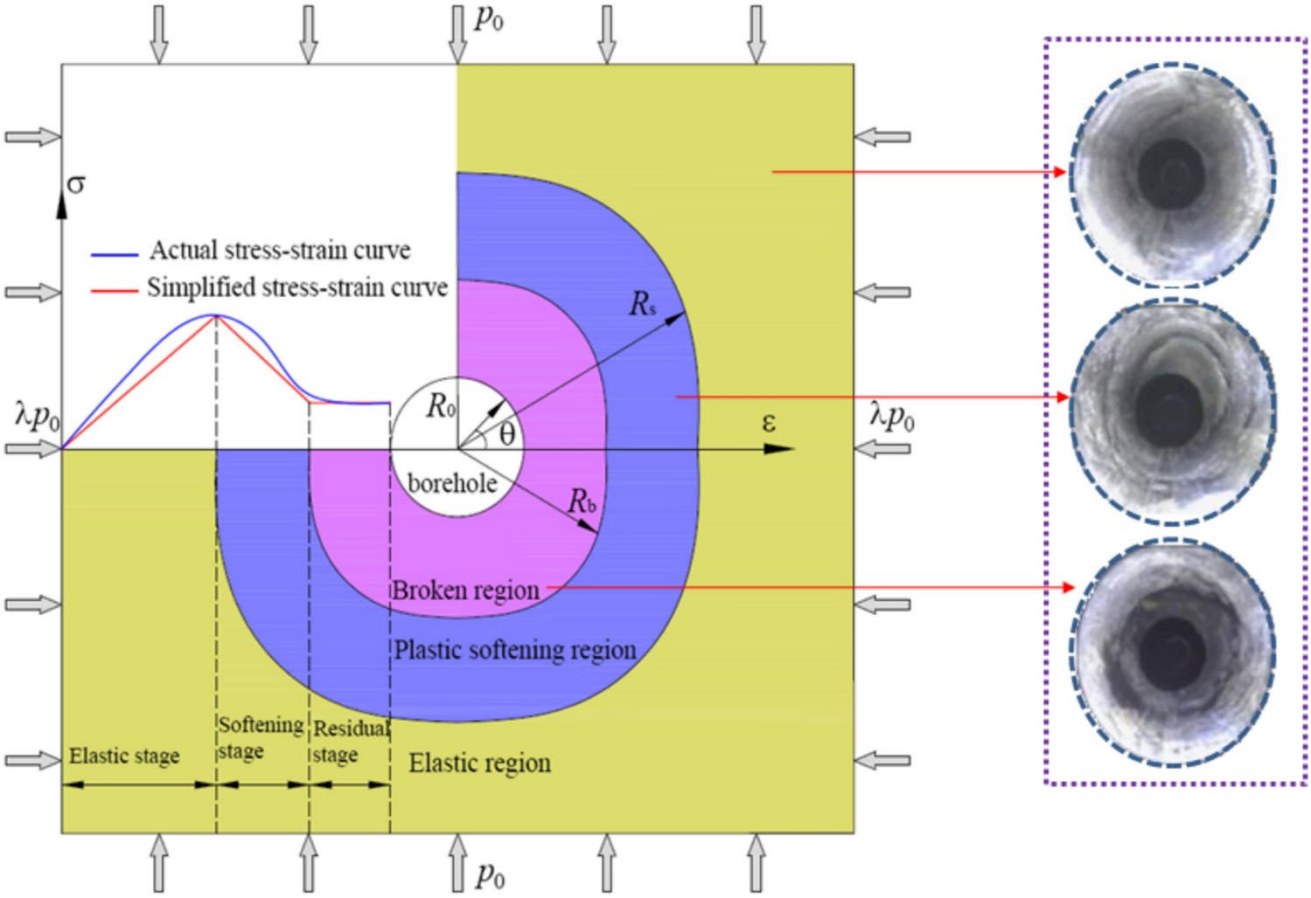
Mechanical model of coal around borehole.

In addition, on the basis of huge amounts of early borehole data, the coal around borehole also can be subdivided into three regions, i.e., broken region (“b”), plastic softening region (“s”) , and elastic region (“e”). Figure 2 is the mechanical model of coal around borehole. In Fig. 2, *λ* is the lateral pressure coefficient, and it can be expressed as the horizontal to vertical stress ratio. *λp*_0_ and *p*_0_ are the horizontal and vertical stress, respectively. *R*_0_ is the radius of the borehole, and *R*_b_ and *R*_s_ are the radii of the broken and plastic softening regions, respectively. *σ* is coal stress, *ε* is coal strain, *θ* is the polar angle in polar coordinate system. In order to simplify calculation, the linearization of complete stress–strain model of coal is curried out, and the simplified constitutive model is divided into three stages. The blue line represents the actual stress–strain relation of coal, and the red line represents the simplified stress–strain relation of coal. From Fig. 2, it can be observed that, in the pre-peak stage, the elastic stress–strain relation is used to analyze the mechanical model of coal around borehole. In the strain-softening stage, a linear strain-softening constitutive relation between stress and plastic strain is adopted. In the broken stage, when the plastic strain satisfies a certain relationship, the coal will break.

### Drucker-Prager (DP) strength criterion

In DP criterion, the intermediate principal stress is considered to reflect the actual stress distribution. Based on the theory of surrounding rock of underground engineering,^1^ the yield function of DP strength criterion under the plane strain during different stress conditions can be expressed as

1 $$f = \sqrt {J_{2} } - K_{i} I_{1} - S_{i}$$

where *I*_1_ is the first stress invariant, *J*_2_ is the second invariant of deviator stress. *σ*_1_, *σ*_2,_ and *σ*_3_ are the maximum principal stress, the intermediate principal stress, and the minimum principal stress, respectively. Thus, *I*_1_ and *J*_2_ can be described as

2 $$\left\{ \begin{gathered} I_{1} = \sigma_{1} + \sigma_{2} + \sigma_{3} \hfill \\ J_{2} = \frac{1}{6}\left[ {\left( {\sigma_{1} - \sigma_{2} } \right)^{2} + \left( {\sigma_{1} - \sigma_{3} } \right)^{2} + \left( {\sigma_{2} - \sigma_{3} } \right)^{2} } \right] \hfill \\ \end{gathered} \right.$$

*K*_*i*_ and *S*_*i*_ are the calculation parameters, their relationship with the cohesion *c* and internal friction angle *φ* can be expressed as

3 $$\left\{ \begin{gathered} K_{i} = \frac{{\sin \varphi_{i} }}{{\sqrt {9 + 3\sin^{2} \varphi_{i} } }} \hfill \\ S_{i} = \frac{{\sqrt 3 c_{i} \cos \varphi_{i} }}{{\sqrt {3 + \sin^{2} \varphi_{i} } }} \hfill \\ \end{gathered} \right.$$

In the process of calculation, the intermediate principal stress coefficient *b* is used to represent a relationship between the principal stresses. And it is given by

4 $$b = \frac{{\sigma_{2} - \sigma_{3} }}{{\sigma_{1} - \sigma_{3} }}$$

According to Eq. (1) and Eq. (4), the DP strength criterion can be rewritten as

5 $$f = \sigma_{1} - M_{i} \sigma_{3} - N_{i}$$

where $$\xi = \sqrt {\frac{{b^{2} - b + 1}}{3}}$$, $$M_{i} = \frac{{\xi + 2K_{i} - bK_{i} }}{{\xi - bK_{i} - K_{i} }}$$, $$N_{i} = \frac{{S_{i} }}{{\xi - bK_{i} - K_{i} }}$$.

Generally, the stress of coal in a polar coordinate system can be expressed as

6 $$\left\{ \begin{gathered} \sigma_{1} { = }\sigma_{\theta } \hfill \\ \sigma_{2} { = }\sigma_{z} \hfill \\ \sigma_{3} { = }\sigma_{r} \hfill \\ \end{gathered} \right.$$

where *σ*_θ_, *σ*_r,_ and *σ*_z_ are the radial, tangential, and axial stress of the coal around borehole, respectively. For convenience of calculations, the yield function of DP strength criterion in different stages can be also expressed as a general form, i.e.,

7 $$f = \sigma_{\theta }^{i} - M_{i} \sigma_{r}^{i} - N_{i}$$

The subscript symbol “*i*” can be replaced by “e”, “s” and “b”.

### Plastic strain-softening model

**Fig. 3:**
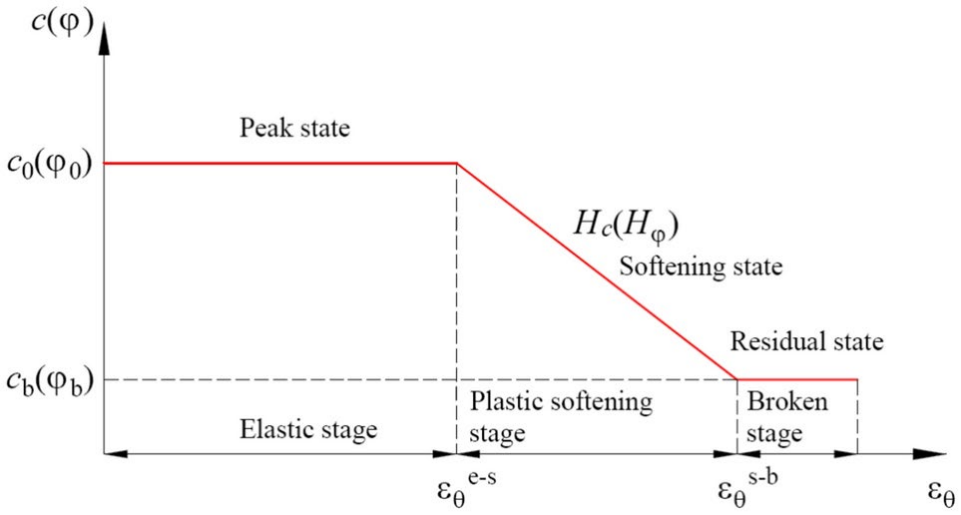
Softening model of strength parameters.

The results of a large amount of tests show that once coal stress exceeds peak strength, the coal rapidly enters the plastic softening stage. Different from the traditional constitutive model, the prominent characteristic of plastic softening is the coal strength decreases with an increase in deformation, until decays to the residual strength, as shown in Fig. 3. Brown et al., Joseph, and Li et al., proposed the methods for estimating the post-peak mechanical behavior of rocks, and verified by the experiments^22,35,36^ Based on these achievements, it is a classical approach to regard the cohesion *c* and the internal friction angle *φ* as the function of strain. Linear softening relationship is the most frequently used method which is easy to achieve analytical solution, and it has become even more attention. Therefore, the softening of coal can be expressed as follows

8 $$\left\{ \begin{gathered} c = c_{0} ,{\kern 1pt} {\kern 1pt} {\kern 1pt} r \ge R_{{\text{s}}} \hfill \\ c = c_{0} - H_{{\text{c}}} \left( {\varepsilon_{\theta }^{{\text{s}}} - \varepsilon_{\theta }^{{\text{e - s}}} } \right),{\kern 1pt} {\kern 1pt} {\kern 1pt} R_{{\text{s}}} \ge r \ge R_{{\text{b}}} \hfill \\ c = c_{{\text{b}}} ,{\kern 1pt} {\kern 1pt} {\kern 1pt} R_{0} \le r \le R_{{\text{b}}} \hfill \\ \end{gathered} \right.$$

9 $$\left\{ \begin{gathered} \varphi = \varphi_{0} ,{\kern 1pt} \;r \ge R_{{\text{s}}} \hfill \\ \varphi = \varphi_{0} - H_{\varphi } \left( {\varepsilon_{\theta }^{{\text{s}}} - \varepsilon_{\theta }^{{\text{e - s}}} } \right),\;R_{{\text{s}}} \ge r \ge R_{{\text{b}}} \hfill \\ \varphi = \varphi_{{\text{b}}} ,{\kern 1pt} \;R_{0} \le r \le R_{{\text{b}}} \hfill \\ \end{gathered} \right.$$

where *c*_0_ and *φ*_0_ are the initial cohesion and initial internal friction angle, respectively; *c*_b_ and *φ*_b_ are the residual cohesion and residual internal friction angle, respectively; *H*_c_ and *H*_φ_ are the softening coefficient of cohesion and internal friction angle in the plastic softening region, respectively; *ε*_*θ*_^s^ is the shear strain of plastic softening region; *ε*_*θ*_^e-s^ is the shear strain at the interface between the elastic and plastic softening region; *ε*_*θ*_^s-b^ is the shear strain at the interface between the broken and plastic softening region.

In Eq. (8) and Eq. (9), *H*_c_ and *H*_φ_ can be expressed as

10 $$\left\{ \begin{gathered} H_{c} = \frac{{c_{0} - c_{{\text{b}}} }}{{\varepsilon_{\theta }^{{\text{s - b}}} - \varepsilon_{\theta }^{{\text{e - s}}} }} \hfill \\ H_{\varphi } = \frac{{\varphi_{0} - \varphi_{{\text{b}}} }}{{\varepsilon_{\theta }^{{\text{s - b}}} - \varepsilon_{\theta }^{{\text{e - s}}} }} \hfill \\ \end{gathered} \right.$$

### Dilatancy model

In the process of plastic deformation, the volume strain changes from compression to expansion before destruction, it is called dilatancy. Based on the theory of plastic potential for material, the equation of dilatancy is given by

11 $${\text{d}}\varepsilon_{ij}^{{\text{p}}} = {\text{d}}\lambda \frac{\partial Q}{{\partial \sigma_{ij} }}$$

where d*ε*_*ij*_^p^ is the plastic strain increment; d*σ*_*ij*_ is the stress increment; *Q* is the plastic potential function; and d*λ* is the plastic factor increment. In this study, the plastic potential function *Q* has the same general form as the yield function *f*. Thus, in the plastic potential function *Q*, replaces internal friction angle *φ* with dilatancy angle *ψ*. The plastic potential function *Q* under different stages can be expressed as

12 $$Q = \sigma_{\theta } - M_{i}^{*} \sigma_{r} - N_{i}^{*}$$

where *M*_i_^*^ and* N*_i_^*^ are the dilatancy coefficients of the plastic softening and broken stage, respectively.

Thus, the solution of plastic strain increment can be obtained as follows by substituting Eq. (12) into Eq. (11):

13 $$\left\{ \begin{gathered} {\text{d}}\varepsilon_{r}^{{\text{p}}} {\text{ = d}}\lambda \frac{\partial Q}{{\partial \sigma_{r} }} = - M_{i}^{*} {\text{d}}\lambda \hfill \\ {\text{d}}\varepsilon_{\theta }^{{\text{p}}} {\text{ = d}}\lambda \frac{\partial Q}{{\partial \sigma_{\theta } }} = {\text{d}}\lambda \hfill \\ \end{gathered} \right.$$

In summary, the relationship between plastic strain increment and dilatancy coefficient by using the linear non-associated flow rule can be written as

14 $$\Delta \varepsilon_{r}^{{\text{p}}} + M_{i}^{*} \Delta \varepsilon_{\theta }^{{\text{p}}} = 0$$

### Elastic–plastic solution of the coal around borehole

#### Basic equations

Regardless of the body load, the coal around borehole can be simplified as a 2D analysis model. Thus, the equilibrium differential equation under three regions in a polar coordinate system can be expressed as

15 $$\frac{{{\text{d}}\sigma_{ri} }}{{{\text{d}}r}} + \frac{{\sigma_{ri} - \sigma_{\theta i} }}{r} = 0$$

where *σ*_*θi*_ and *σ*_*ri*_ are the tangential and radial stress in three regions, respectively.

Based on the elastic–plastic theory, the geometric equation of the model can be written as

16 $$\left\{ \begin{gathered} \varepsilon_{ri} { = }\frac{{{\text{d}}u_{i} }}{{{\text{d}}r}} \hfill \\ \varepsilon_{\theta i} { = }\frac{{u_{i} }}{r} \hfill \\ \end{gathered} \right.$$

where *ε*_*θi*_ and *ε*_*ri*_ are the tangential and radial strain in three regions, respectively. *u*_i_ is the displacement in three regions.

It’s worth noting that the deformation of coal generated by origin stress has been completed during the geological evolvement process, and the relative deformation will occur in the process of drilling. Thus, the stress increment should be adopted in the constitutive equation, it can be expressed as

17 $$\left\{ \begin{gathered} \varepsilon_{ri} { = }\frac{{1 - \upsilon^{2} }}{E}\left( {\Delta \sigma_{ri} - \frac{\upsilon }{1 - \upsilon }\Delta \sigma_{\theta i} } \right) \hfill \\ \varepsilon_{\theta i} { = }\frac{{1 - \upsilon^{2} }}{E}\left( {\Delta \sigma_{\theta i} - \frac{\upsilon }{1 - \upsilon }\Delta \sigma_{ri} } \right) \hfill \\ \end{gathered} \right.$$

where ∆*σ*_*ri*_ and ∆*σ*_*θi*_ are the radial and tangential stress increments in different regions, respectively. *E* is the elastic modulus of coal and *υ* is Poisson’s ratio of coal.

#### Solution of the elastic region

**Fig. 4:**
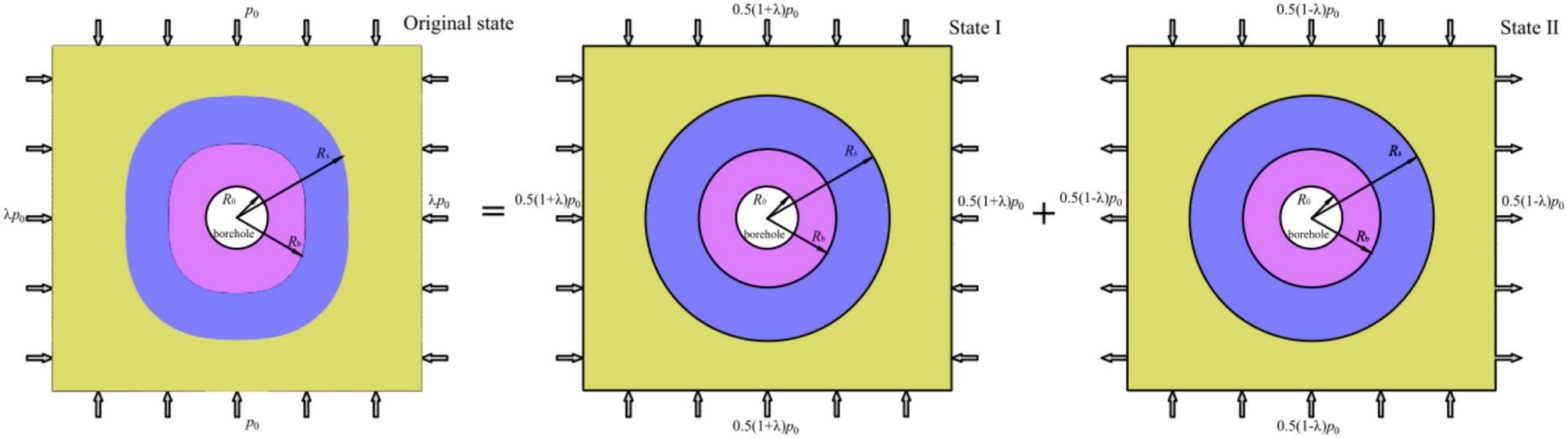
Decomposition model of coal under different stress conditions.

The coal stress field around borehole under different stress conditions can be disassembled into two conditions, as shown in Fig. 4. In state I, the coal is loaded with a uniformly distributed pressure, and the value of pressure is 0.5(1 + λ) *p*_0_. In state II, the coal is loaded a vertical pressure and horizontal tension, and the values are all 0.5(1-*λ*) *p*_0_.

In state I, according to the theory of elasticity, the stress can be expressed as

18 $$\left\{ \begin{gathered} \sigma_{r} { = }\frac{{\left( {1 + \lambda } \right)p_{0} }}{2}\left( {1 - \frac{{R_{{\text{s}}}^{2} }}{{r^{2} }}} \right) + \sigma_{r}^{{R_{{\text{s}}} }} \frac{{R_{{\text{s}}}^{2} }}{{r^{2} }} \hfill \\ \sigma_{\theta } = \frac{{\left( {1 + \lambda } \right)p_{0} }}{2}\left( {1 + \frac{{R_{{\text{s}}}^{2} }}{{r^{2} }}} \right) - \sigma_{r}^{{R_{{\text{s}}} }} \frac{{R_{{\text{s}}}^{2} }}{{r^{2} }} \hfill \\ \end{gathered} \right.$$

where *σ*_*r*_^*Rs*^ is the radial stress on the interface between the elastic and plastic softening region.

In state II, with Airy stress function, the stress under the polar coordinate system can be written as

19 $$\left\{ \begin{gathered} \sigma_{r} { = } - \frac{{\left( {1 - \lambda } \right)p_{0} }}{2}\left( {1 - 4\frac{{R_{{\text{s}}}^{2} }}{{r^{2} }} + 3\frac{{R_{{\text{s}}}^{4} }}{{r^{4} }}} \right)\cos 2\theta \hfill \\ \sigma_{\theta } = \frac{{\left( {1 - \lambda } \right)p_{0} }}{2}\left( {1 + 3\frac{{R_{{\text{s}}}^{4} }}{{r^{4} }}} \right)\cos 2\theta \hfill \\ \end{gathered} \right.$$

In summary, the coal stress field around borehole can be obtained by superimposing Eq. (18) and Eq. (19), and the expression is given by

20 $$\left\{ \begin{gathered} \sigma_{r}^{{\text{e}}} { = }\frac{{\left( {1 + \lambda } \right)p_{0} }}{2}\left( {1 - \frac{{R_{{\text{s}}}^{2} }}{{r^{2} }}} \right) + \sigma_{r}^{{R_{{\text{s}}} }} \frac{{R_{{\text{s}}}^{2} }}{{r^{2} }} - \frac{{\left( {1 - \lambda } \right)p_{0} }}{2}\left( {1 - 4\frac{{R_{{\text{s}}}^{2} }}{{r^{2} }} + 3\frac{{R_{{\text{s}}}^{4} }}{{r^{4} }}} \right)\cos 2\theta \hfill \\ \sigma_{\theta }^{{\text{e}}} = \frac{{\left( {1 + \lambda } \right)p_{0} }}{2}\left( {1 + \frac{{R_{{\text{s}}}^{2} }}{{r^{2} }}} \right) - \sigma_{r}^{{R_{{\text{s}}} }} \frac{{R_{{\text{s}}}^{2} }}{{r^{2} }} + \frac{{\left( {1 - \lambda } \right)p_{0} }}{2}\left( {1 + 3\frac{{R_{{\text{s}}}^{4} }}{{r^{4} }}} \right)\cos 2\theta \hfill \\ \end{gathered} \right.$$

Based on the stress continuous condition of the interface between the elastic and plastic softening region, i.e., $\sigma_{r}^{{\text{e}}} = \left. {\sigma_{r}^{{R_{{\text{s}}} }} } \right|_{{r = R_{{\text{s}}} }}$, the tangential stress of the elastic region can be derived as

21 $$\sigma_{\theta }^{{\text{e}}} = \left( {1 + \lambda } \right)p_{0} - \sigma_{r}^{{R_{{\text{s}}} }} + 2\left( {1 - \lambda } \right)p_{0} \cos 2\theta$$

Then, the radial stress can be obtained by substituting Eq. (21) into Eq. (7), as

22 $$\sigma_{r}^{{R_{{\text{s}}} }} = \frac{{\left( {1 + \lambda } \right)p_{0} + 2\left( {1 - \lambda } \right)p_{0} \cos 2\theta - N_{{\text{e}}} }}{{M_{{\text{e}}} }}$$

According to the calculation model in Fig. 2, the origin stress of coal can be expressed as

23 $$\left\{ \begin{gathered} \sigma_{r}^{0} = \frac{{\left( {1 + \lambda } \right)p_{0} }}{2} - \frac{{\left( {1 - \lambda } \right)p_{0} }}{2}\cos 2\theta \hfill \\ \sigma_{\theta }^{0} = \frac{{\left( {1 + \lambda } \right)p_{0} }}{2} + \frac{{\left( {1 - \lambda } \right)p_{0} }}{2}\cos 2\theta \hfill \\ \end{gathered} \right.$$

where *σ*_*r*_^0^ and *σ*_*θ*_^0^ are the radial and tangential origin stress of coal, respectively.

The coal stress increment of the elastic region can be obtained by Eq. (20) subtracting from Eq. (23), as

24 $$\left\{ \begin{gathered} \Delta \sigma_{r}^{{\text{e}}} { = }\sigma_{r}^{{\text{e}}} - \sigma_{0} = - \frac{{\left( {1 + \lambda } \right)p_{0} }}{2}\frac{{R_{{\text{s}}}^{2} }}{{r^{2} }} + \sigma_{r}^{{R_{{\text{s}}} }} \frac{{R_{{\text{s}}}^{2} }}{{r^{2} }} - \frac{{\left( {1 - \lambda } \right)p_{0} }}{2}\left( {3\frac{{R_{{\text{s}}}^{4} }}{{r^{4} }} - 4\frac{{R_{{\text{s}}}^{2} }}{{r^{2} }}} \right)\cos 2\theta \hfill \\ \Delta \sigma_{\theta }^{{\text{e}}} = \sigma_{\theta }^{{\text{e}}} - \sigma_{0} = \frac{{\left( {1 + \lambda } \right)p_{0} }}{2}\frac{{R_{{\text{s}}}^{2} }}{{r^{2} }} - \sigma_{r}^{{R_{{\text{s}}} }} \frac{{R_{{\text{s}}}^{2} }}{{r^{2} }} + \frac{{\left( {1 - \lambda } \right)p_{0} }}{2}\left( {3\frac{{R_{{\text{s}}}^{4} }}{{r^{4} }}} \right)\cos 2\theta \hfill \\ \end{gathered} \right.$$

Then, the coal strain of the elastic region can be obtained by substituting Eq. (24) into Eq. (17), as

25 $$\left\{ \begin{gathered} \varepsilon_{r}^{{\text{e}}} { = }\frac{1 + \upsilon }{E}\left[ { - \frac{{\left( {1 + \lambda } \right)}}{2}p_{0} \frac{{R_{{\text{s}}}^{{2}} }}{{r^{2} }} + \sigma_{r}^{{R_{{\text{s}}} }} \frac{{R_{{\text{s}}}^{{2}} }}{{r^{2} }} - \frac{{\left( {1 - \lambda } \right)}}{2}p_{0} \left( {3\frac{{R_{{\text{s}}}^{4} }}{{r^{4} }} - 4\frac{{R_{{\text{s}}}^{{2}} }}{{r^{2} }}{ + }4\upsilon \frac{{R_{{\text{s}}}^{{2}} }}{{r^{2} }}} \right)\cos 2\theta } \right] \hfill \\ \varepsilon_{\theta }^{{\text{e}}} { = }\frac{1 + \upsilon }{E}\left[ {\frac{{\left( {1 + \lambda } \right)}}{2}p_{0} \frac{{R_{{\text{s}}}^{{2}} }}{{r^{2} }} - \sigma_{r}^{{R_{{\text{s}}} }} \frac{{R_{{\text{s}}}^{{2}} }}{{r^{2} }} + \frac{{\left( {1 - \lambda } \right)}}{2}p_{0} \left( {3\frac{{R_{{\text{s}}}^{4} }}{{r^{4} }} - 4\upsilon \frac{{R_{{\text{s}}}^{{2}} }}{{r^{2} }}} \right)\cos 2\theta } \right] \hfill \\ \end{gathered} \right.$$

Thus, using the geometric equation Eq. (16), the displacement of the elastic region can be calculated as

26 $$u_{{\text{e}}} = \varepsilon_{\theta }^{{\text{e}}} r = \frac{1 + \upsilon }{E}r\left[ {\frac{{\left( {1 + \lambda } \right)}}{2}p_{0} \frac{{R_{{\text{s}}}^{{2}} }}{{r^{2} }} - \sigma_{r}^{{R_{{\text{s}}} }} \frac{{R_{{\text{s}}}^{{2}} }}{{r^{2} }} + \frac{{\left( {1 - \lambda } \right)}}{2}p_{0} \left( {3\frac{{R_{{\text{s}}}^{4} }}{{r^{4} }} - 4\upsilon \frac{{R_{{\text{s}}}^{{2}} }}{{r^{2} }}} \right)\cos 2\theta } \right]$$

#### Solution of the plastic softening region

Based on the stress continuous condition, i.e., $\sigma_{r}^{{\text{e}}} = \left. {\sigma_{r}^{{R_{{\text{s}}} }} } \right|_{{r = R_{{\text{s}}} }}$, the stress of the plastic softening region can be obtained by substituting Eq. (7) into Eq. (15), as

27 $$\left\{ \begin{gathered} \sigma_{r}^{{\text{s}}} { = }\left( {\sigma_{r}^{{R_{{\text{s}}} }} + \frac{{N_{{\text{s}}} }}{{M_{{\text{s}}} - 1}}} \right)\left( {\frac{r}{{R_{{\text{s}}} }}} \right)^{{M_{{\text{s}}} - 1}} - \frac{{N_{{\text{s}}} }}{{M_{{\text{s}}} - 1}} \hfill \\ \sigma_{\theta }^{{\text{s}}} = M_{{\text{s}}} \left( {\sigma_{r}^{{R_{{\text{s}}} }} + \frac{{N_{{\text{s}}} }}{{M_{{\text{s}}} - 1}}} \right)\left( {\frac{r}{{R_{{\text{s}}} }}} \right)^{{M_{{\text{s}}} - 1}} - \frac{{N_{{\text{s}}} }}{{M_{{\text{s}}} - 1}} \hfill \\ \end{gathered} \right.$$

According to elastic-plasticity theory, the strain of the plastic softening region includes two parts, one part is the elastic strain and another part is the plastic strain. So, the strain can be written as

28 $$\left\{ \begin{gathered} \varepsilon_{r}^{{\text{s}}} { = }\varepsilon_{r}^{{{\text{se}}}} + \varepsilon_{r}^{{{\text{sp}}}} \hfill \\ \varepsilon_{\theta }^{{\text{s}}} { = }\varepsilon_{\theta }^{{{\text{se}}}} + \varepsilon_{\theta }^{{{\text{sp}}}} \hfill \\ \end{gathered} \right.$$

The superscript symbols “se” and “sp” represent the elastic and plastic strain in the plastic softening region, respectively.

Based on the dilatancy model, the strain of the plastic softening region can be expressed as

29 $$g = \varepsilon_{r}^{{\text{s}}} + M_{{\text{s}}}^{*} \varepsilon_{\theta }^{{\text{s}}}$$

Bringing Eq. (28) into Eq. (29), as

30 $$\begin{gathered} g = \varepsilon_{r}^{{{\text{se}}}} + \varepsilon_{r}^{{{\text{sp}}}} + M_{{\text{s}}}^{*} \left( {\varepsilon_{\theta }^{{{\text{se}}}} + \varepsilon_{\theta }^{{{\text{sp}}}} } \right) = \left( {\varepsilon_{r}^{{{\text{se}}}} + M_{{\text{s}}}^{*} \varepsilon_{\theta }^{{{\text{se}}}} } \right) + \left( {\varepsilon_{r}^{{{\text{sp}}}} + M_{{\text{s}}}^{*} \varepsilon_{\theta }^{{{\text{sp}}}} } \right) \hfill \\ {\kern 1pt} {\kern 1pt} {\kern 1pt} {\kern 1pt} {\kern 1pt} {\kern 1pt} {\kern 1pt} {\kern 1pt} {\kern 1pt} {\kern 1pt} = \left( {\varepsilon_{r}^{{{\text{se}}}} + M_{{\text{s}}}^{*} \varepsilon_{\theta }^{{{\text{se}}}} } \right) \hfill \\ \end{gathered}$$

The elastic strain in the plastic softening region can be regarded as a constant, and its value should be equal to the elastic strain at the interface between the elastic and plastic softening region. Thus, the stress continuous condition can be expressed as $\varepsilon_{r}^{{{\text{se}}}} = \left. {\varepsilon_{r}^{{\text{e}}} } \right|_{{r = R_{{\text{s}}} }}$ and $\varepsilon_{\theta }^{{{\text{se}}}} = \left. {\varepsilon_{\theta }^{{\text{e}}} } \right|_{{r = R_{{\text{s}}} }}$. Combining the geometric equation Eq. (16), it can be derived as

31 $$\begin{gathered} g = \varepsilon_{r}^{{{\text{se}}}} + M_{{\text{s}}}^{*} \varepsilon_{\theta }^{{{\text{se}}}} { = }\left. {\varepsilon_{r}^{{\text{e}}} } \right|_{{r = R_{{\text{s}}} }} + M_{{\text{s}}}^{*} \left. {\varepsilon_{\theta }^{{\text{e}}} } \right|_{{r = R_{{\text{s}}} }} \hfill \\ {\kern 1pt} {\kern 1pt} {\kern 1pt} {\kern 1pt} {\kern 1pt} {\kern 1pt} {\kern 1pt} {\kern 1pt} {\kern 1pt} = \frac{{{\text{d}}u_{s} }}{{{\text{d}}r}} + M_{{\text{s}}}^{*} \frac{{u_{s} }}{r} \hfill \\ \end{gathered}$$

At the interface between the elastic and plastic softening region, the displacement should be satisfied with the continuity condition, i.e., $u_{{\text{s}}} = \left. {u_{{\text{e}}} } \right|_{{r = R_{{\text{s}}} }}$. Thus, the displacement solution of Eq. (31) can be obtained through integration as

32 $$u_{{\text{s}}} = \frac{Ar}{{M_{{\text{s}}}^{*} + 1}}\left[ {1 - \left( {\frac{{R_{{\text{s}}} }}{r}} \right)^{{M_{{\text{s}}}^{*} + 1}} } \right] + Br\left( {\frac{{R_{{\text{s}}} }}{r}} \right)^{{M_{{\text{s}}}^{*} + 1}}$$

The coefficients *A* and *B* can be expressed as follows

$$\left\{ \begin{gathered} B{ = }\frac{1 + \upsilon }{E}\left[ {\frac{{\left( {1 + \lambda } \right)}}{2}p_{0} - \sigma_{r}^{{R_{{\text{s}}} }} + \frac{{\left( {1 - \lambda } \right)}}{2}p_{0} \left( {3 - 4\upsilon } \right)\cos 2\theta } \right] \hfill \\ A = \frac{1 + \upsilon }{E}\left\{ {\frac{{\left( {1 + \lambda } \right)}}{2}p_{0} \left( {M_{{\text{s}}}^{*} - 1} \right) - \sigma_{r}^{{R_{{\text{s}}} }} \left( {M_{{\text{s}}}^{*} - 1} \right) + \frac{{\left( {1 - \lambda } \right)}}{2}p_{0} \left[ {M_{{\text{s}}}^{*} \left( {3 - 4\upsilon } \right) - \left( {4\upsilon - 1} \right)} \right]\cos 2\theta } \right\} \hfill \\ \end{gathered} \right.$$

The strain of the plastic softening region can be deduced by substituting Eq. (32) into the geometric equation Eq. (16), as follows

33 $$\left\{ \begin{gathered} \varepsilon_{r}^{{\text{s}}} { = }\frac{A}{{M_{{\text{s}}}^{*} + 1}}\left[ {1 + M_{{\text{s}}}^{*} \left( {\frac{{R_{{\text{s}}} }}{r}} \right)^{{M_{{\text{s}}}^{*} + 1}} } \right] - M_{{\text{s}}}^{*} B\left( {\frac{{R_{{\text{s}}} }}{r}} \right)^{{M_{{\text{s}}}^{*} + 1}} \hfill \\ \varepsilon_{\theta }^{{\text{s}}} { = }\frac{A}{{M_{{\text{s}}}^{*} + 1}}\left[ {1 - \left( {\frac{{R_{{\text{s}}} }}{r}} \right)^{{M_{{\text{s}}}^{*} + 1}} } \right] + B\left( {\frac{{R_{{\text{s}}} }}{r}} \right)^{{M_{{\text{s}}}^{*} + 1}} \hfill \\ \end{gathered} \right.$$

#### Solution of the broken region

According to the similar computation process, when the stress continuous condition as $\left. {\sigma_{r}^{b} } \right|_{{r = R_{0} }} = 0$, the stress of the broken region can be obtained by substituting Eq. (7) into Eq. (15), as

34 $$\left\{ \begin{gathered} \sigma_{r}^{{\text{b}}} { = }\frac{{N_{{\text{b}}} }}{{M_{{\text{b}}} - 1}}\left[ {\left( {\frac{r}{{R_{0} }}} \right)^{{M_{{\text{b}}} - 1}} - 1} \right] \hfill \\ \sigma_{\theta }^{{\text{b}}} = \frac{{M_{{\text{b}}} N_{{\text{b}}} }}{{M_{{\text{b}}} - 1}}\left[ {\left( {\frac{r}{{R_{0} }}} \right)^{{M_{{\text{b}}} - 1}} - 1} \right] + N_{{\text{b}}} \hfill \\ \end{gathered} \right.$$

In broken region, the strain of coal also consists of two parts, as follows

35 $$\left\{ \begin{gathered} \varepsilon_{r}^{{\text{b}}} { = }\varepsilon_{r}^{{{\text{be}}}} + \varepsilon_{r}^{{{\text{bp}}}} \hfill \\ \varepsilon_{\theta }^{{\text{b}}} { = }\varepsilon_{\theta }^{{{\text{be}}}} + \varepsilon_{\theta }^{{{\text{bp}}}} \hfill \\ \end{gathered} \right.$$

The superscript symbols “be” and “bp” represent the elastic and plastic strain in the broken region, respectively.

Based on the dilatancy model, the strain of the broken region can be expressed as

36 $$g = \varepsilon_{r}^{{\text{b}}} + M_{{\text{b}}}^{*} \varepsilon_{\theta }^{{\text{b}}} = \varepsilon_{r}^{{{\text{be}}}} + M_{{\text{b}}}^{*} \varepsilon_{\theta }^{{{\text{be}}}}$$

Similarly, the elastic strain in the broken region can be regarded as a constant, and its value should be equal to the plastic strain at the interface between the broken and plastic softening region. So, the stress continuous condition can be expressed as $\varepsilon_{r}^{{{\text{be}}}} = \left. {\varepsilon_{r}^{{\text{s}}} } \right|_{{r = R_{{\text{b}}} }}$ and $\varepsilon_{\theta }^{{{\text{be}}}} = \left. {\varepsilon_{\theta }^{{\text{s}}} } \right|_{{r = R_{{\text{b}}} }}$. Then, the geometric equation of the broken region can be written as

37 $$\begin{gathered} g = \varepsilon_{r}^{{{\text{be}}}} + M_{{\text{b}}}^{*} \varepsilon_{\theta }^{{{\text{be}}}} { = }\left. {\varepsilon_{r}^{{\text{s}}} } \right|_{{r = R_{0} }} + M_{s}^{*} \left. {\varepsilon_{\theta }^{{\text{s}}} } \right|_{{r = R_{0} }} \hfill \\ {\kern 1pt} {\kern 1pt} {\kern 1pt} {\kern 1pt} {\kern 1pt} {\kern 1pt} {\kern 1pt} {\kern 1pt} {\kern 1pt} = \frac{{{\text{d}}u_{b} }}{{{\text{d}}r}} + M_{{\text{b}}}^{*} \frac{{u_{b} }}{r} \hfill \\ \end{gathered}$$

The displacement also should be satisfied the continuity condition, i.e., $u_{{\text{b}}} = \left. {u_{{\text{s}}} } \right|_{{r = R_{{\text{b}}} }}$, then the solution of the broken region can be derived as

38 $$u_{{\text{b}}} = \frac{Kr}{{M_{{\text{b}}}^{*} + 1}}\left[ {1 - \left( {\frac{{R_{{\text{b}}} }}{r}} \right)^{{M_{{\text{b}}}^{*} + 1}} } \right] + \frac{Ar}{{M_{{\text{s}}}^{*} + 1}}\left[ {1 - \left( {\frac{{R_{{\text{s}}} }}{{R_{{\text{b}}} }}} \right)^{{M_{{\text{s}}}^{*} + 1}} } \right]\left( {\frac{{R_{{\text{b}}} }}{r}} \right)^{{M_{{\text{b}}}^{*} }} + BR_{{\text{b}}} \left( {\frac{{R_{{\text{s}}} }}{{R_{{\text{b}}} }}} \right)^{{M_{{\text{s}}}^{*} + 1}} \left( {\frac{{R_{{\text{b}}} }}{r}} \right)^{{M_{{\text{b}}}^{*} }}$$

The coefficient *K* can be expressed as

$$K = \frac{A}{{M_{{\text{s}}}^{*} + 1}}\left[ {\left( {1 + M_{{\text{b}}}^{*} } \right) + \left( {M_{{\text{s}}}^{*} - M_{{\text{b}}}^{*} } \right)\left( {\frac{{R_{{\text{s}}} }}{{R_{{\text{b}}} }}} \right)^{{M_{{\text{s}}}^{*} + 1}} } \right] + \left( {M_{{\text{b}}}^{*} - M_{{\text{s}}}^{*} } \right)B\left( {\frac{{R_{{\text{s}}} }}{{R_{{\text{b}}} }}} \right)^{{M_{{\text{s}}}^{*} + 1}}$$

The strain in the broken region can be also determined by substituting Eq. (38) into the geometric equation Eq. (16), as follows

39 $$\left\{ \begin{gathered} \varepsilon_{r}^{{\text{s}}} { = }\frac{K}{{M_{{\text{b}}}^{*} + 1}}\left[ {1 + M_{{\text{b}}}^{*} \left( {\frac{{R_{{\text{b}}} }}{r}} \right)^{{M_{{\text{b}}}^{*} + 1}} } \right] + \frac{A}{{M_{{\text{s}}}^{*} + 1}}\left( {1 - M_{{\text{b}}}^{*} } \right)\left[ {1 - \left( {\frac{{R_{{\text{s}}} }}{{R_{{\text{b}}} }}} \right)^{{M_{{\text{s}}}^{*} + 1}} } \right]\left( {\frac{{R_{{\text{b}}} }}{r}} \right)^{{M_{{\text{b}}}^{*} }} - M_{{\text{b}}}^{*} B\left( {\frac{{R_{{\text{s}}} }}{{R_{{\text{b}}} }}} \right)^{{M_{{\text{s}}}^{*} + 1}} \left( {\frac{{R_{{\text{b}}} }}{r}} \right)^{{M_{{\text{b}}}^{*} + 1}} \hfill \\ \varepsilon_{\theta }^{{\text{s}}} { = }\frac{K}{{M_{{\text{b}}}^{*} + 1}}\left[ {1 - \left( {\frac{{R_{{\text{b}}} }}{r}} \right)^{{M_{{\text{b}}}^{*} + 1}} } \right] + \frac{A}{{M_{{\text{s}}}^{*} + 1}}\left[ {1 - \left( {\frac{{R_{{\text{s}}} }}{{R_{{\text{b}}} }}} \right)^{{M_{{\text{s}}}^{*} + 1}} } \right]\left( {\frac{{R_{{\text{b}}} }}{r}} \right)^{{M_{{\text{b}}}^{*} }} + B\left( {\frac{{R_{{\text{s}}} }}{{R_{{\text{b}}} }}} \right)^{{M_{{\text{s}}}^{*} + 1}} \left( {\frac{{R_{{\text{b}}} }}{r}} \right)^{{M_{{\text{b}}}^{*} + 1}} \hfill \\ \end{gathered} \right.$$

#### Solution of the radii (*R*_s_ and *R*_b_)

Based on the stress continuous condition at the interface between the broken and plastic softening region, i.e., $\sigma_{r}^{{\text{s}}} = \left. {\sigma_{r}^{{\text{b}}} } \right|_{{r = R_{{\text{b}}} }}$, it can be obtained a relationship between *R*_s_ and *R*_0_ as follows

40 $$\frac{{R_{{\text{s}}} }}{{R_{0} }} = \left[ {1 + \frac{{\sigma_{r}^{{R_{{\text{s}}} }} \left( {M_{{\text{b}}} - 1} \right)}}{{N_{{\text{b}}} }}} \right]^{{\frac{1}{{M_{{\text{b}}} - 1}}}}$$

At the interface between the broken and plastic softening region, the cohesion and internal friction angle all attenuate to the residual value, i.e., $C_{{\text{s}}} = \left. {C_{{\text{b}}} } \right|_{{r = R_{{\text{b}}} }}$ and $\varphi_{{\text{s}}} = \left. {\varphi_{{\text{b}}} } \right|_{{r = R_{{\text{b}}} }}$. Using the softening coefficient *H*_c_ and *H*_φ_, a relationship between *R*_s_ and *R*_b_ can be derived as follows

41 $$\left\{ \begin{gathered} \frac{{R_{{\text{s}}} }}{{R_{{\text{b}}} }} = \left[ {1 + \frac{{c_{0} - c_{b} }}{{H_{c} }}\frac{{M_{{\text{s}}}^{*} + 1}}{{B\left( {M_{{\text{s}}}^{*} + 1} \right) - A}}} \right]^{{\frac{1}{{M_{{\text{s}}}^{*} + 1}}}} \hfill \\ \frac{{R_{{\text{s}}} }}{{R_{{\text{b}}} }} = \left[ {1 + \frac{{\varphi_{0} - \varphi_{b} }}{{H_{\varphi } }}\frac{{M_{{\text{s}}}^{*} + 1}}{{B\left( {M_{{\text{s}}}^{*} + 1} \right) - A}}} \right]^{{\frac{1}{{M_{{\text{s}}}^{*} + 1}}}} \hfill \\ \end{gathered} \right.$$

In summary, the radii (*R*_s_ and *R*_b_) can be expressed as

42 $$\left\{ \begin{gathered} R_{{\text{s}}} = R_{0} \left[ {1 + \frac{{\sigma_{r}^{{R_{{\text{s}}} }} \left( {M_{{\text{b}}} - 1} \right)}}{{N_{{\text{b}}} }}} \right]^{{\frac{1}{{M_{{\text{b}}} - 1}}}} \hfill \\ R_{{\text{b}}} = R_{0} \left[ {1 + \frac{{\sigma_{r}^{{R_{{\text{s}}} }} \left( {M_{{\text{b}}} - 1} \right)}}{{N_{{\text{b}}} }}} \right]^{{\frac{1}{{M_{{\text{b}}} - 1}}}} /\left[ {1 + \frac{{c_{0} - c_{b} }}{{H_{c} }}\frac{{M_{{\text{s}}}^{*} + 1}}{{B\left( {M_{{\text{s}}}^{*} + 1} \right) - A}}} \right]^{{\frac{1}{{M_{{\text{s}}}^{*} + 1}}}} \hfill \\ R_{{\text{b}}} = R_{0} \left[ {1 + \frac{{\sigma_{r}^{{R_{{\text{s}}} }} \left( {M_{{\text{b}}} - 1} \right)}}{{N_{{\text{b}}} }}} \right]^{{\frac{1}{{M_{{\text{b}}} - 1}}}} /\left[ {1 + \frac{{\varphi_{0} - \varphi_{b} }}{{H_{\varphi } }}\frac{{M_{{\text{s}}}^{*} + 1}}{{B\left( {M_{{\text{s}}}^{*} + 1} \right) - A}}} \right]^{{\frac{1}{{M_{{\text{s}}}^{*} + 1}}}} \hfill \\ \end{gathered} \right.$$

## Analysis of results

### Example

To investigate the evolution law of stress and deformation of coal around pneumatic drill borehole, the Taoyuan coal mine, as the engineering background employs in this study. The average horizontal stress is 24.0 MPa, and the average vertical stress is 20.0 MPa. The coefficient of lateral pressure is *λ* = 1.2, and the radius of borehole is *R*_0_ = 0.05 m. The coal mechanical parameters are as follows: *E* = 1050 MPa, *υ* = 0.3, *c*_0_ = 3 MPa, *c*_b_ = 0.6 MPa, *φ*_0_ = 30°, *φ*_b_ = 20°, *ψ* = 10°, *H*_c_ = 430 MPa and *H*_φ_ = 1500°^16,18^

### Analysis of influence parameters

#### Analysis of the influences of lateral pressure coefficient

**Fig. 5:**
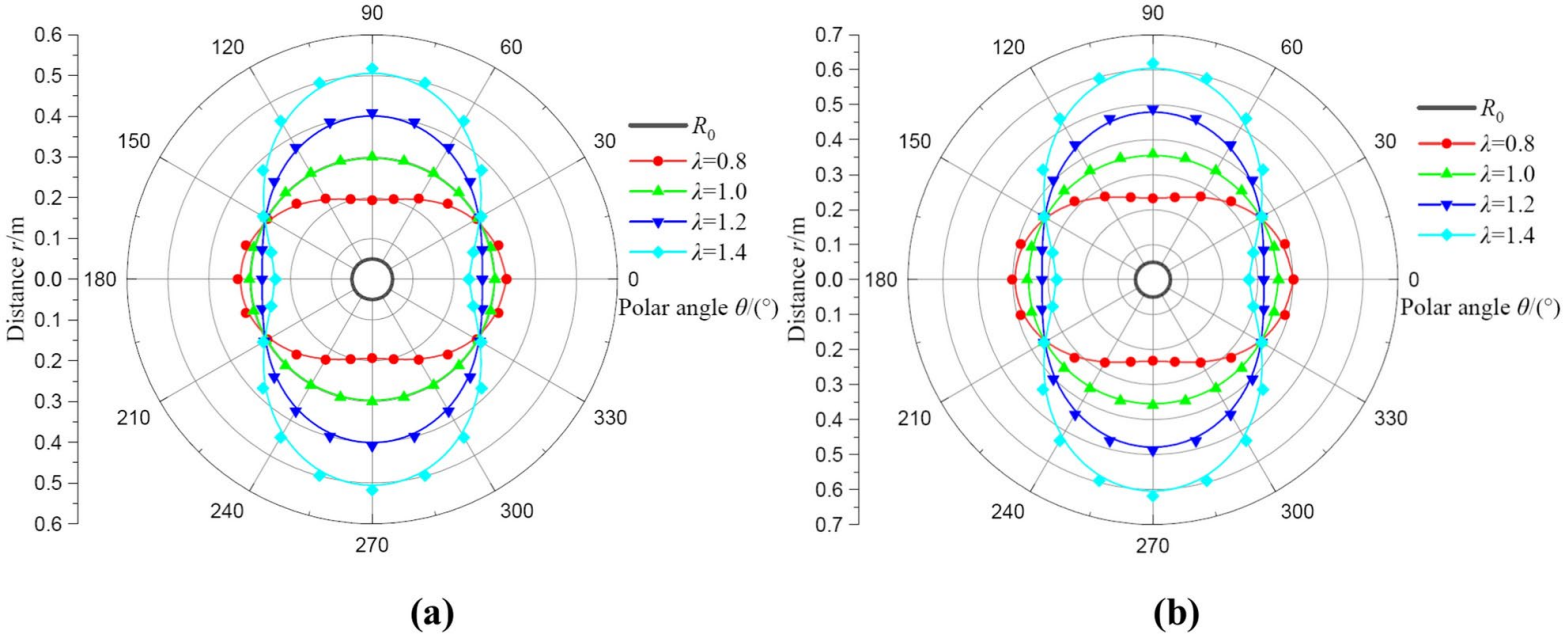
The deformation distributions of broken and plastic softening regions under different lateral pressure coefficients. (**a**) result of* R*_b_; (**b**) result of* R*_s_.

A large amount of literature suggests that the value of *λ* is generally between 0.8 and 1.5^1–5^ Hence, it is very necessary to find the relationship between the lateral pressure coefficient and deformation. In this part, the value of *λ* is selected as 0.8, 1.0, 1.2, and 1.4, and other parameters remain the same. The deformation distributions of two regions with different *λ* are shown in Fig. 5. When *λ* = 1.0, the shape of the broken and plastic softening region is circular, and the radii are *R*_b_ = 0.30 m and *R*_s_ = 0.36 m, respectively. When *λ* > 1.0, it represents the horizontal stress is bigger than vertical stress, the sides of borehole occurs a significant depression and the top–bottom of borehole are damaged most seriously. Additionally, based on the results of the first quadrant (0 ~ *π*/2), the radii of two regions almost increase linearity gradually with the increased polar angle, which means that the larger polar angle is, the larger deformation zone becomes. Also, notice that the deformation degree of the sides of borehole decreases not significantly as the lateral pressure coefficient increases, but the results of the top–bottom of borehole are opposite. This result will seriously attenuate the safety of the top–bottom of borehole. However, When *λ* < 1.0, all results are the contrary. Therefore, the lateral pressure coefficient *λ* plays an essential important role in the deformation of coal around borehole under different stress conditions.

**Fig. 6:**
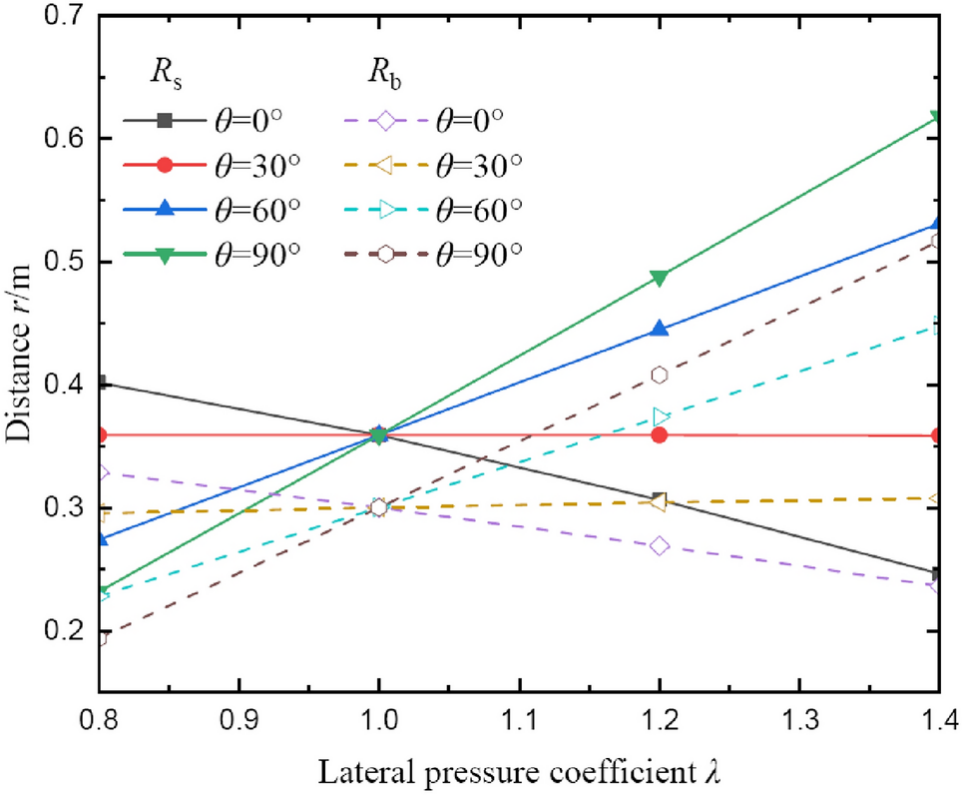
The radii of broken and plastic softening regions under different lateral pressure coefficients.

Figure 6 illustrates the effects of lateral pressure coefficient on radii of broken and plastic softening regions. The responses of radii with lateral pressure coefficient comply with different laws in a range of polar angle 0 ~ *π*/2. It can be observed that there is a boundary value for the polar angle that corresponds to the variation law of radii in different regions, and the value is approximately *θ* = 30°. When *θ* > 30°, the radii increase with the increase of lateral pressure coefficient, and vice versa. So, in the first quadrant, *θ* = 30° is the inflection point of the distribution of two regions, and it is also confirmed by Fig. 5. It can further explain that the deformation of coal around borehole is non-uniform under different stress conditions, which is favorable for predicting the distribution of broken and plastic softening regions. Moreover, when *θ* = 30°, no matter how the lateral pressure coefficient λ changes, the radii of two regions will remain unchanged. Also notice that when the vertical load stress remains constant, the radii of two regions on the side of borehole are inversely related to the horizontal stress. But the radii of two regions on the top–bottom of borehole are positively correlated with the horizontal stress.

#### Analysis of the influences of vertical stress

**Fig. 7:**
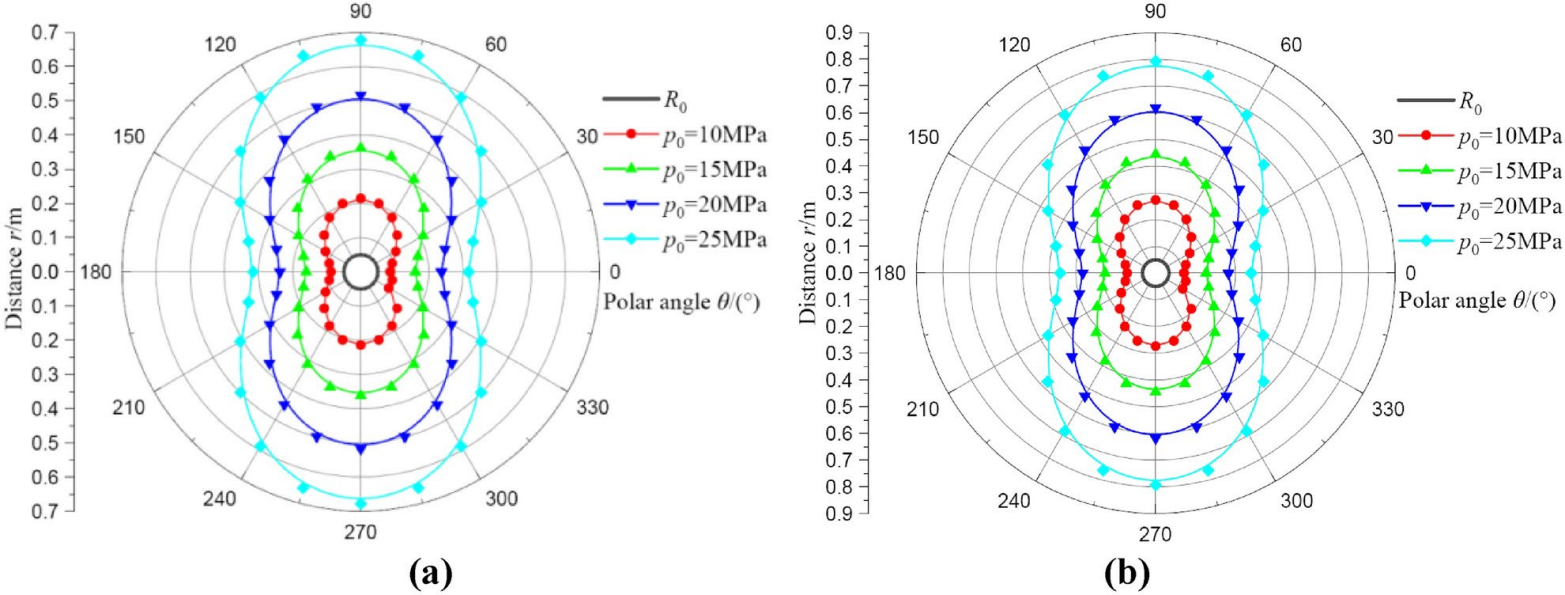
The deformation distributions of broken and plastic softening regions under different vertical stress. (a) result of* R*_b_; (b) result of* R*_s_.

The influences of vertical stress on deformation distributions of broken and plastic softening regions are shown in Fig. 7. In a calculating process, the range and value are *p*_0_ = 10 ~ 25 MPa, *λ* = 1.4. The radii of broken and plastic softening regions increase significantly with an increase in vertical stress, and the distribution of two regions is similar to the peanut shape. Besides that, a vertical stress change has little affected on the distribution shape of two regions, and only affects on the value of radii. Therefore, the shape of two regions can be obtained as long as the lateral pressure coefficient is determined. The key factor that affects the shape of broken and plastic softening regions is the lateral pressure coefficient, and the distribution of two regions will gradually possess the peanut shape from a circular shape with lateral pressure coefficient *λ* changes from 1.0 to 1.4.

**Fig. 8:**
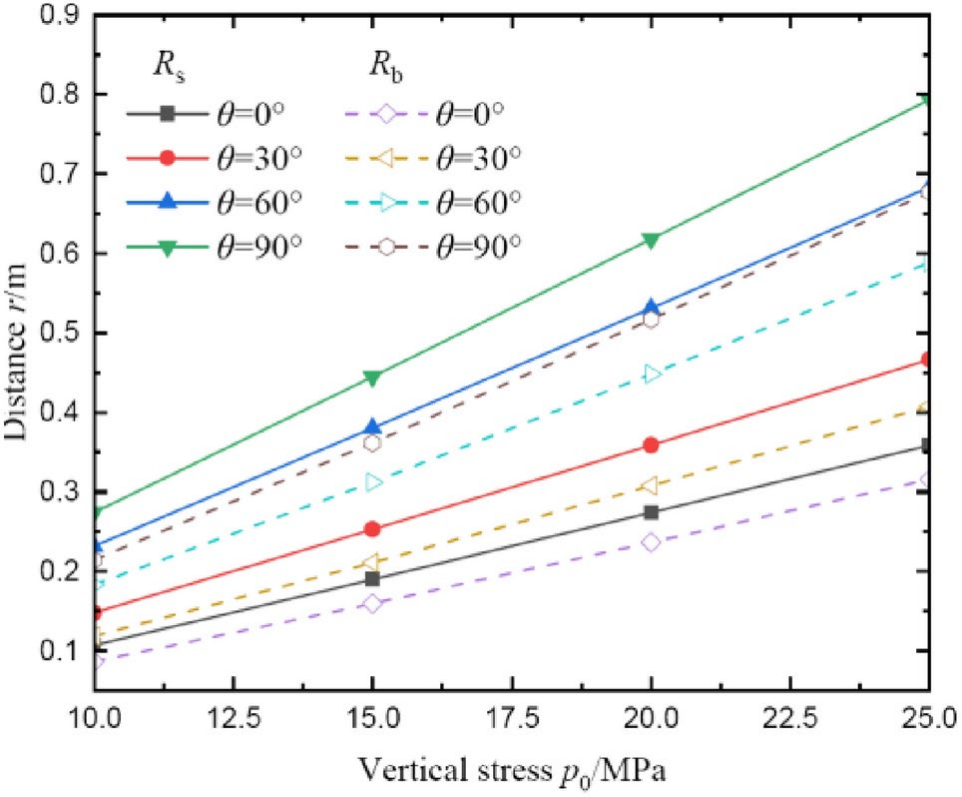
The radii of broken and plastic softening regions under different vertical stress.

Figure 8 shows the influences of vertical stress on radii of broken and plastic softening regions. It can be seen that the radii of broken and plastic softening regions not only increase linear with the increase of vertical stress, but also the gradient of radii increment in two regions are increased early and decreased afterward with the increase of polar angle. *θ* = 60° is the inflection point of the gradient of radii increment. For instance, when the input polar angle changes from 0° to 90°, the slopes of *R*_s_ curves are 16.79%, 21.21%, 30.13%, and 33.42%, respectively. Besides that, when the polar angle is *θ* = 90°, the radii of two regions are the largest. And at *θ* = 0°, the radii are smallest.

#### Analysis of the influences of borehole radius

**Fig. 9:**
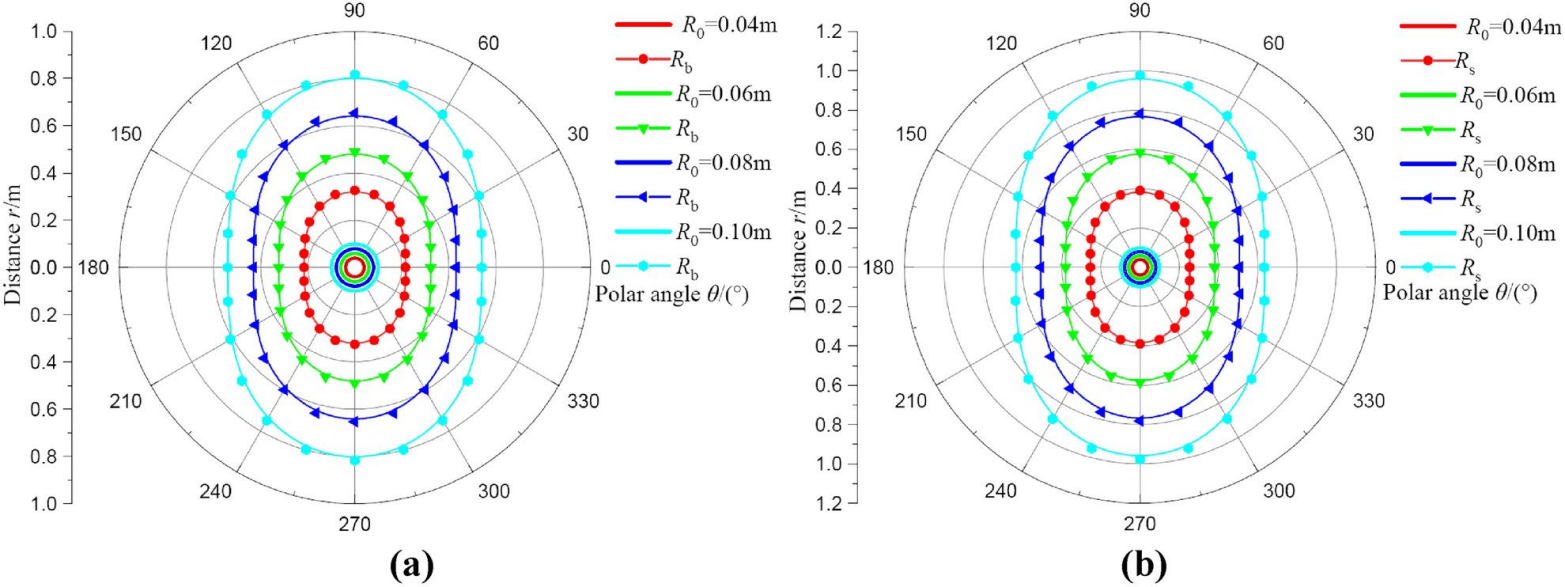
The deformation distributions of broken and plastic softening regions under different borehole radii. (**a**) result of* R*_b_; (**b**) result of* R*_s_.

Figure 9 represents the influences of borehole radius on deformation distributions of broken and plastic softening regions. Here, the varying range of parameters is *R*_0_ = 0.04 ~ 0.1 m, *λ* = 1.2, and *p*_0_ = 20 MPa. As shown in Fig. 9, the larger the borehole radius, the greater range of the broken region and plastic softening region. Under the same condition, the smaller the borehole radius results in a better safety of coal around borehole. Therefore, the small diameter stressometer is preferred for monitoring the coal stress, to attenuate the adverse impacts on the coal stress field.

**Fig.10:**
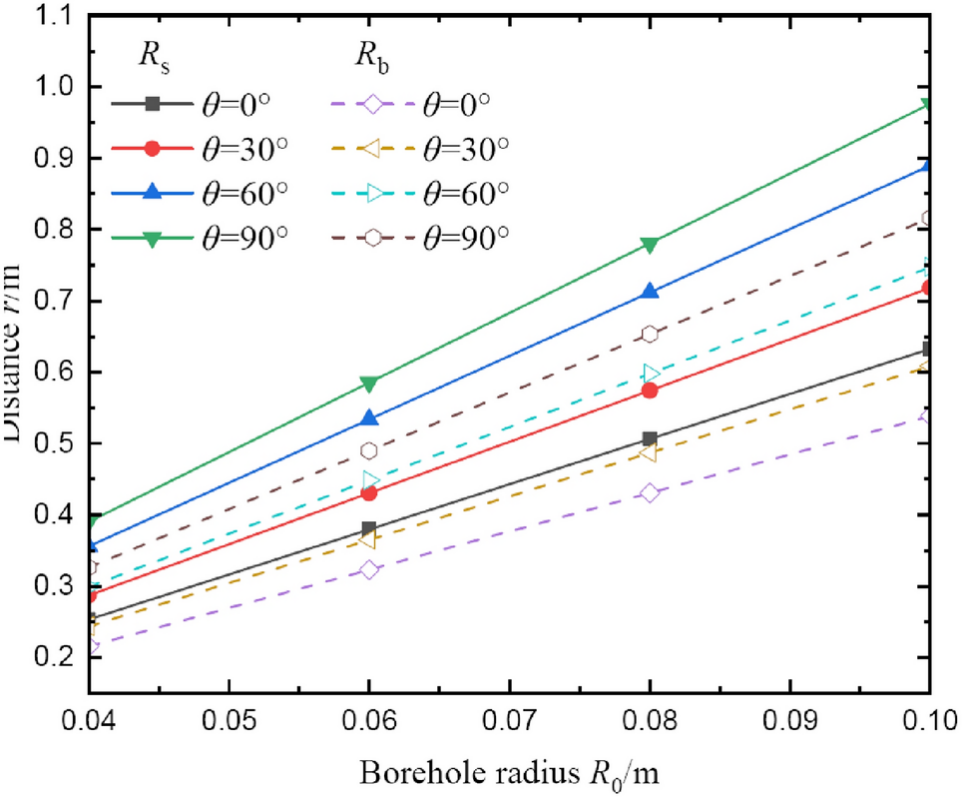
The radii of broken and plastic softening regions under different borehole size.

Figure 10 shows the influences of borehole size on radii of broken and plastic softening regions. The results indicate the variation law of radii in broken and plastic softening regions under different borehole sizes is similar to the results of the influence of vertical stress. The radii in two regions also increase linearly with the increase in borehole size. Similar to the effects of vertical stress, *θ* = 60° is also the inflection point of the gradient of radii increment.

#### Analysis of the influences of intermediate principal stress coefficient

**Fig. 11:**
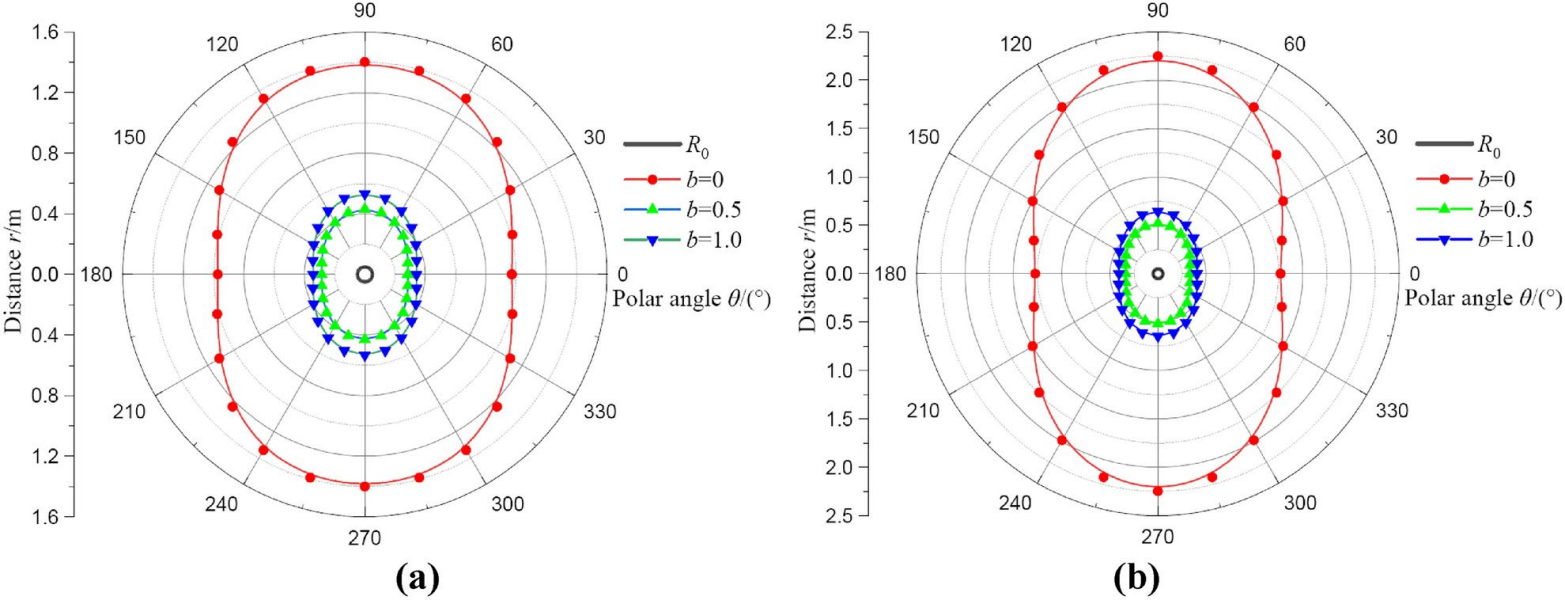
The deformation distributions of broken and plastic softening regions under different intermediate principal stress coefficients. (**a**) result of* R*_b_; (**b**) result of* R*_s_.

Figure 11 expresses the influences of intermediate principal stress coefficient on deformation distributions of broken and plastic softening regions. In this part, the value of *b* is selected as 0, 0.5, and 1.0, *λ* = 1.2, and *p*_0_ = 20 MPa. From Fig. 11, it can be seen that the intermediate principal stress coefficient has nearly no effect on the distribution shape of two regions, a smaller intermediate principal stress coefficient indicates a larger range of deformation. It’s important to note that the larger intermediate principal stress coefficient does not necessarily mean the smaller range of deformation. When the coefficient *b* = 0.5, the deformation distribution of two regions is the smallest. For instance, when *b* = 0.5, the *R*_s_ and *R*_b_ values at *θ* = 0° are 0.28 m and 0.33 m, respectively. However, when *b* = 1.0, the *R*_s_ and *R*_b_ values at *θ* = 0° are 0.34 m and 0.40 m, respectively. The values of *R*_s_ and *R*_b_ have risen, not fallen. Thus, the effect of intermediate principal stress coefficient on the calculated results of coal around borehole is also a critical factor. Meanwhile, considering the influence of the intermediate principal stress coefficient, the calculating results will be more correct.

**Fig. 12:**
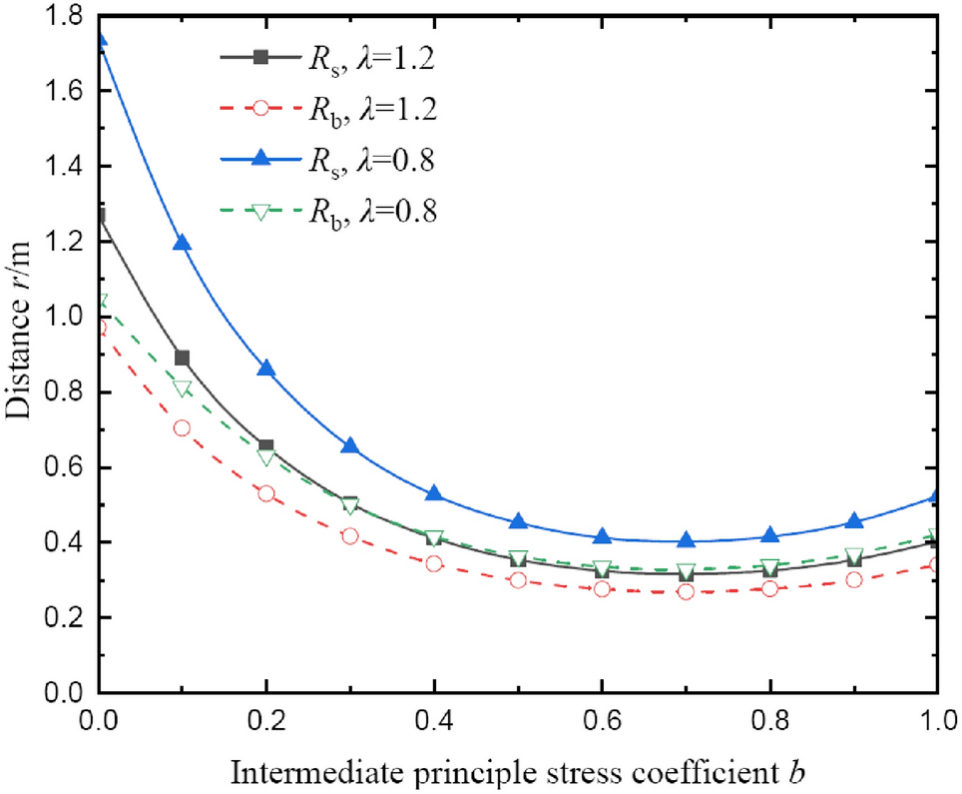
The radii of broken and plastic softening regions under different intermediate principal stress coefficients.

The effects of intermediate principal stress coefficient on radii of broken and plastic softening regions at *θ* = 0° are depicted in Fig. 12. The radii of two regions first decrease as the intermediate principal stress coefficient is increased and then increase as the intermediate principal stress coefficient is further increased. When *b* decreases from 0.4 to 0, the influence of the intermediate principal stress coefficient increases abruptly, and after that, the change is relatively smooth. At *b* = 0, the radii of two regions are the largest. When *b* = 0, i.e., *σ*_r_ = *σ*_z_, it represents the influence of intermediate principal stress is ignored, which is equivalent to the uniaxial compression. When *b* = 1.0, i.e., *σ*_θ_ = *σ*_z_, it is equivalent to the biaxial compression. The intermediate principal stress can reflect the influence of coal strength and deformation at a certain extent, therefore, considering the influence of intermediate principal stress is more reasonable for the calculation in the stress field of coal around borehole. In addition, interestingly enough, there is almost an optimal value for the intermediate principal stress coefficient corresponding to the minimum radii of two regions, and the value is approximately *b* = 0.7. That result will be of great significance to predict the stress and deformation of coal around borehole at different stress conditions. Additionally, the mechanism of the intermediate principal stress coefficient also requires to be verified by the true triaxial test, this is the focus of future research.

#### Analysis of the influences of cohesion

**Fig. 13:**
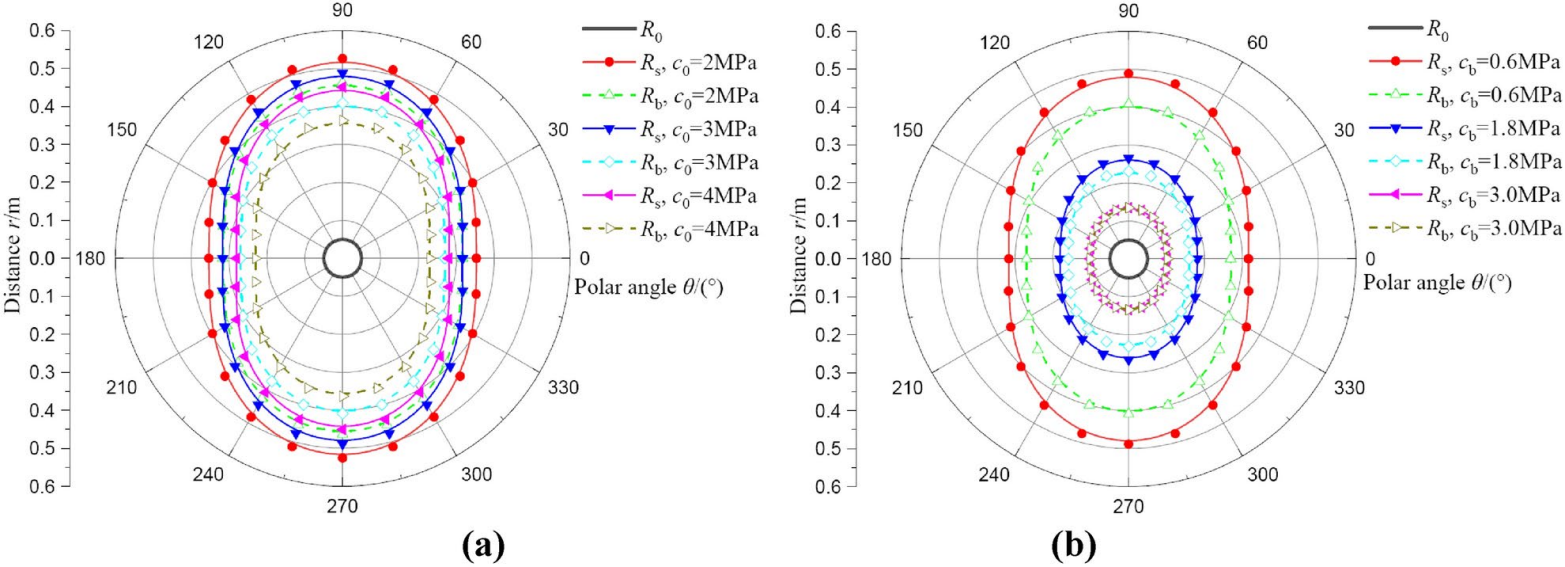
The deformation distributions of broken and plastic softening regions under different cohesion. (**a**) result of initial cohesion; (**b**) result of residual cohesion.

In this section, cohesion includes two parts, one is initial cohesion, and another is residual cohesion. For initial cohesion, the value of *c*_0_ is selected as 2.0, 3.0, and 4.0 MPa, other parameters are *c*_b_ = 0.6 MPa, *λ* = 1.2, and *p*_0_ = 20 MPa, result as shown in Fig. 13a. For residual cohesion, the value of *c*_b_ is selected as 0.6, 1.8, and 3.0 MPa, other parameters are *c*_0_ = 3.0 MPa, *λ* = 1.2, and *p*_0_ = 20 MPa, result as shown in Fig. 13b. It is obvious that the distribution of two regions decreases with an increase in initial and residual cohesion, and these parameters do not seem to have affected the shape of two regions. For Fig. 13b, when *c*_b_ = 3.0 MPa, i.e., *c*_b_ = *c*_0_, the distribution size of broken and plastic softening region is coincident. The initial and residual cohesion are usually determined by experiments, but the strength of these parameters generally decreases in practice, because the disturbances of borehole must be dealt with in construction. If the plastic strain softening is not considered, a question of less estimation of the deformation in two regions will exist, resulting in a large safety loss. As it should be, the residual cohesion also can be increased according to grouting in construction to improve the safety of coal around borehole.

**Fig. 14:**
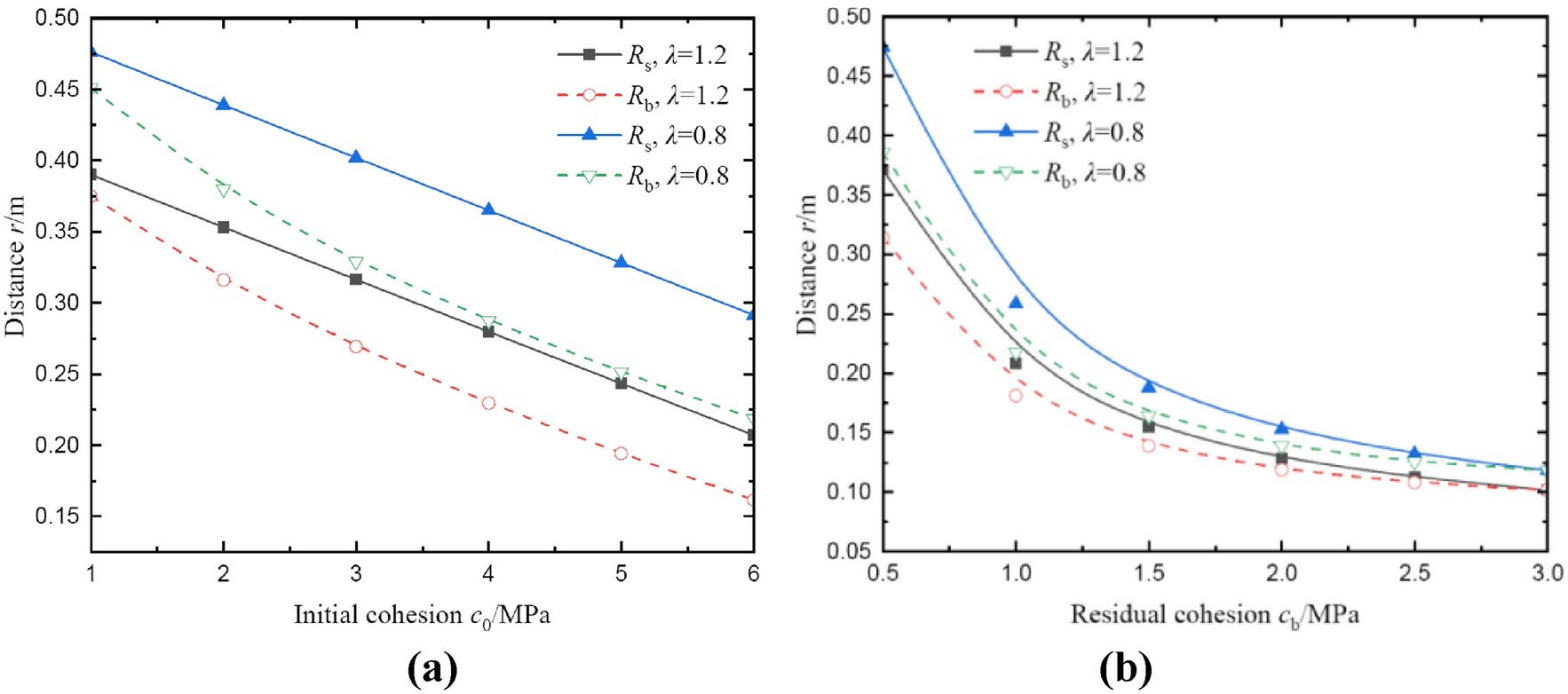
The radii of broken and plastic softening regions under different cohesion. (**a**) result of initial cohesion; (**b**) result of residual cohesion.

Figure 14 shows the effects of initial and residual cohesion on radii of broken and plastic softening regions at *θ* = 0°. In Fig. 14a, the radius of plastic softening region decreases linearly with increases in initial cohesion, but the radius of broken region reduces nonlinearly. This nonlinear effect is slight, and it is negligible in a simplified analysis. For example, *c*_0_ = 1.0 ~ 6.0 MPa, *λ* = 1.2, the radius of broken region decreases by 52.89% (from 0.38 m to 0.16 m), and the radius of plastic softening region decreases by 46.15% (from 0.39 m to 0.21 m). In Fig. 14b, it can be seen that the radii of two regions all decrease nonlinearly with increases in residual cohesion, and the decrease of radii also gradually declines and finally tends to be stable. For instance, *c*_b_ = 0.5 ~ 3.0 MPa, *λ* = 1.2, the radius of broken region decreases by 64.52% (from 0.31 m to 0.11 m), and the radius of plastic softening region decreases by 70.27% (from 0.37 m to 0.11 m). Therefore, the coal will be more safe and stable by increases in cohesion.

#### Analysis of the influences of internal friction angle

**Fig. 15:**
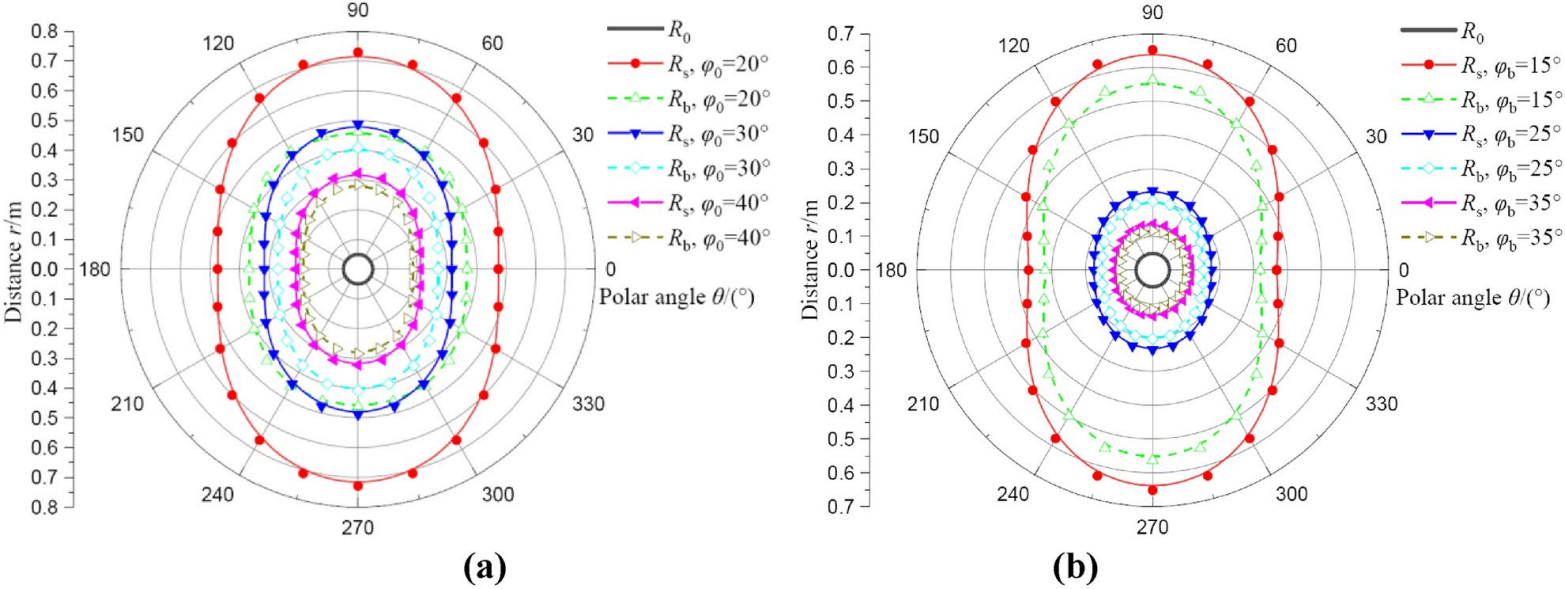
The deformation distributions of broken and plastic softening regions under different internal friction angle. (**a**) result of initial internal friction angle; (**b**) result of residual internal friction angle.

Figure 15 represents the influences of initial and residual internal friction angle on deformation distributions of broken and plastic softening regions at *θ* = 0°. For the initial internal friction angle, the value of *φ*_0_ is selected as 20, 30, and 40°, result as shown in Fig. 15a. For the residual internal friction angle, the value of *φ*_b_ is selected as 15, 25, and 35°, resulting as shown in Fig. 15b. It can be seen that the distribution of two regions increases with initial and residual internal friction angle decrease, and this result is consistent with the effects of cohesion. The residual internal friction angle is also a very important parameter for the radii of two regions and has attracted more attention in academic arguments^16–18^ This view also deserves a further explanation, in this study, for example, *φ*_b_ = 15 ~ 35°, the radius of broken region decreases by 70.27% (from 0.37 m to 0.11 m), and the radius of plastic softening region decreases by 71.87% (from 0.32 m to 0.09 m).

**Fig. 16:**
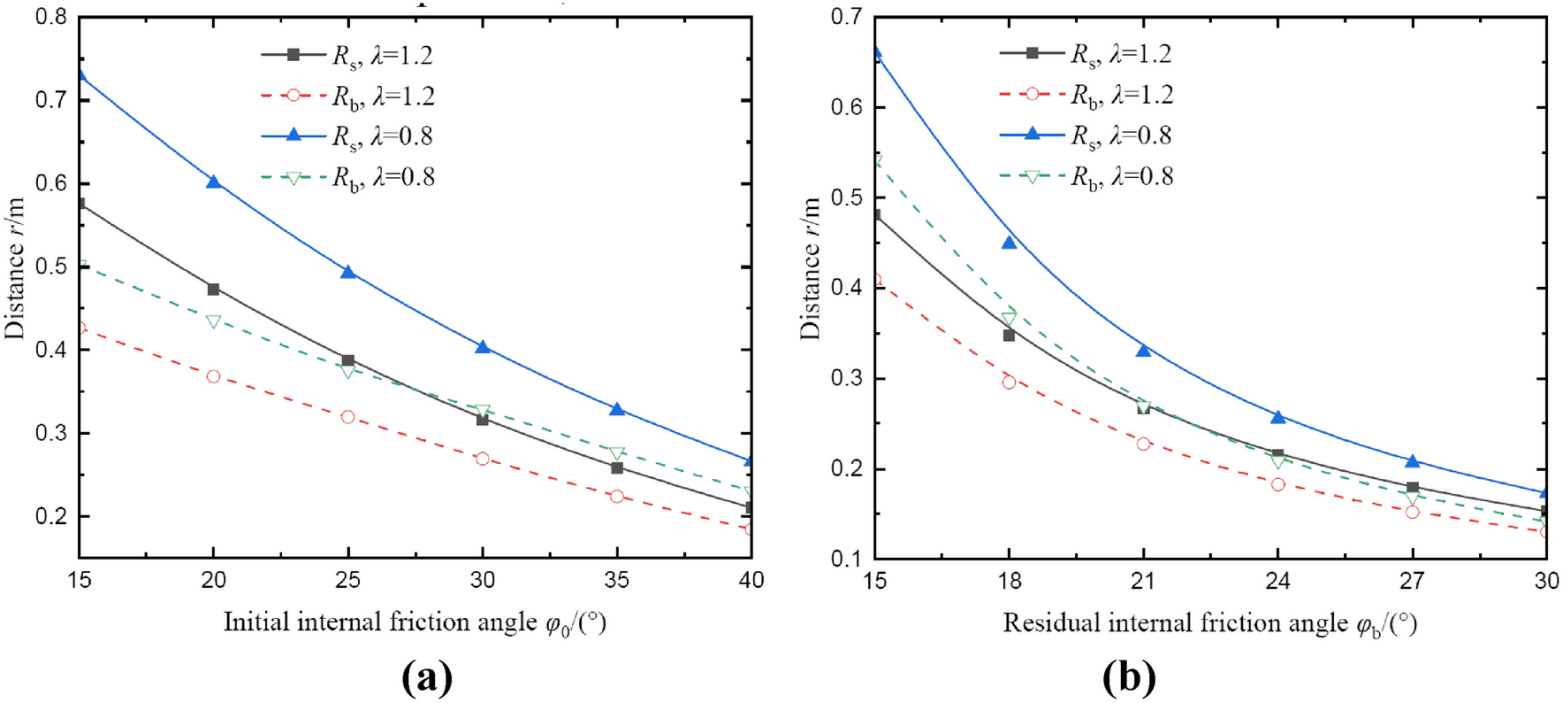
The radii of broken and plastic softening regions under different internal friction angle. (**a**) result of initial internal friction angle; (**b**) result of residual internal friction angle.

Figure 16 shows the effects of initial and residual internal friction on radii of broken and plastic softening regions at *θ* = 0°. It can be seen that from Fig. 16a, the radius of plastic softening region reduces nonlinearly with increases in initial internal friction angle, and this nonlinear effect is also slight. Additionally, it also can be seen that the radii of two regions all decrease nonlinearly with increases in residual internal friction angle and these effects are quite similar to the effects of residual cohesion. To avoid repetition, it’s not described in this section.

## Numerical validation

**Fig. 17:**
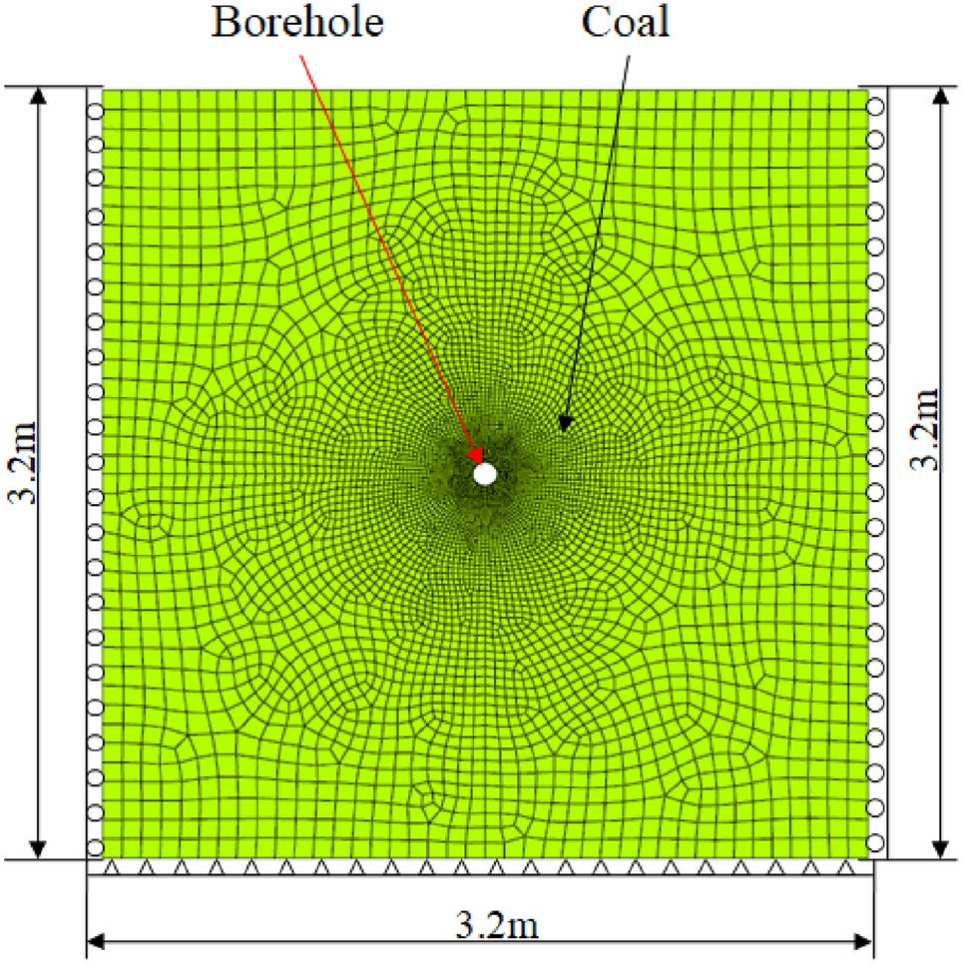
Finite element model and boundary conditions of coal around borehole.

In order to validate the rationality of this present analytical method, a two-dimensional finite element analysis of the coal around borehole under different stress conditions is conducted by using the numerical analysis software (ANSYS). The coal around borehole is seen as an isotropic material, the geometric model is square and the side length is 3.2 × 3.2 m. A borehole with a diameter of 0.10 m is located in the middle of the geometric model, as shown in Fig. 17. The numerical model is considered a plane strain problem and applies the 8-noded quadrilateral elements to determine the deformation and stress of coal. The meshing criterion is assessed by comparing the deformation distribution in the final results, when after increasing the element by 10%, the maximum deformation value changed by less than 1%, and then the meshing is believed reasonable. Additionally, local mesh refinement around the borehole is conducted in the numerical model. The boundary conditions in the numerical model are as follows: the bottom of the model is set as a fully fixed constraint, and both sides of the model are set as horizontal fixed hinge support, and the top of the model is freedom. By the geometric dimensions, the side length of coal is thirty times more than borehole diameter, which is beneficial for reducing the influence of boundary.

**Fig. 18:**
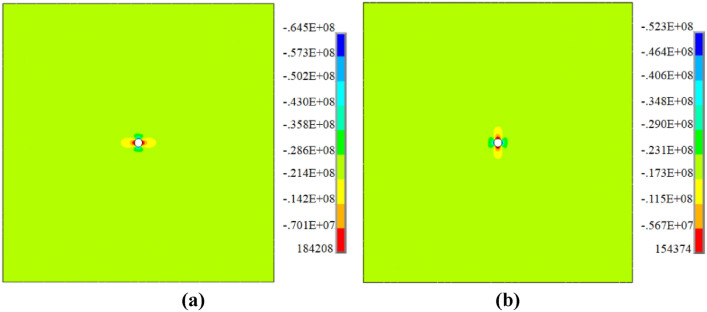
Stress results of finite element method (unit: Pa). (**a**) result of x direction; (**b**) result of y direction.

In the finite element method (FEM), the constitutive model is an elastic-perfectly plastic stress–strain model with DP strength criterion, so the plastic softening relation will be redefined and implanted in the original numerical procedure, and other conditions are the same as the analytical method. According to Eq. (8) and Eq. (10), the softening parameters are introduced into the numerical model referring to the relevant studies^37,38^ Figure 18 represents the stress distribution of coal around borehole (x and y direction). It can be seen that the stress concentration occurs around borehole and is accompanied by the large deformation. In the same section, the distance from borehole is bigger, and the stress distribution is more uniform. Therefore, the identification of drilling affects regions is one of the fundamental steps, as well as the key point of stress analysis of coal around borehole, and it is dependent on many factors, such as lateral pressure coefficient, original stress, borehole radius, intermediate principal stress coefficient, internal friction angle, and cohesion, etc. The influences of these parameters on different regions are all discussed in detail in the analytical method, and are not presented again in here.

**Fig. 19:**
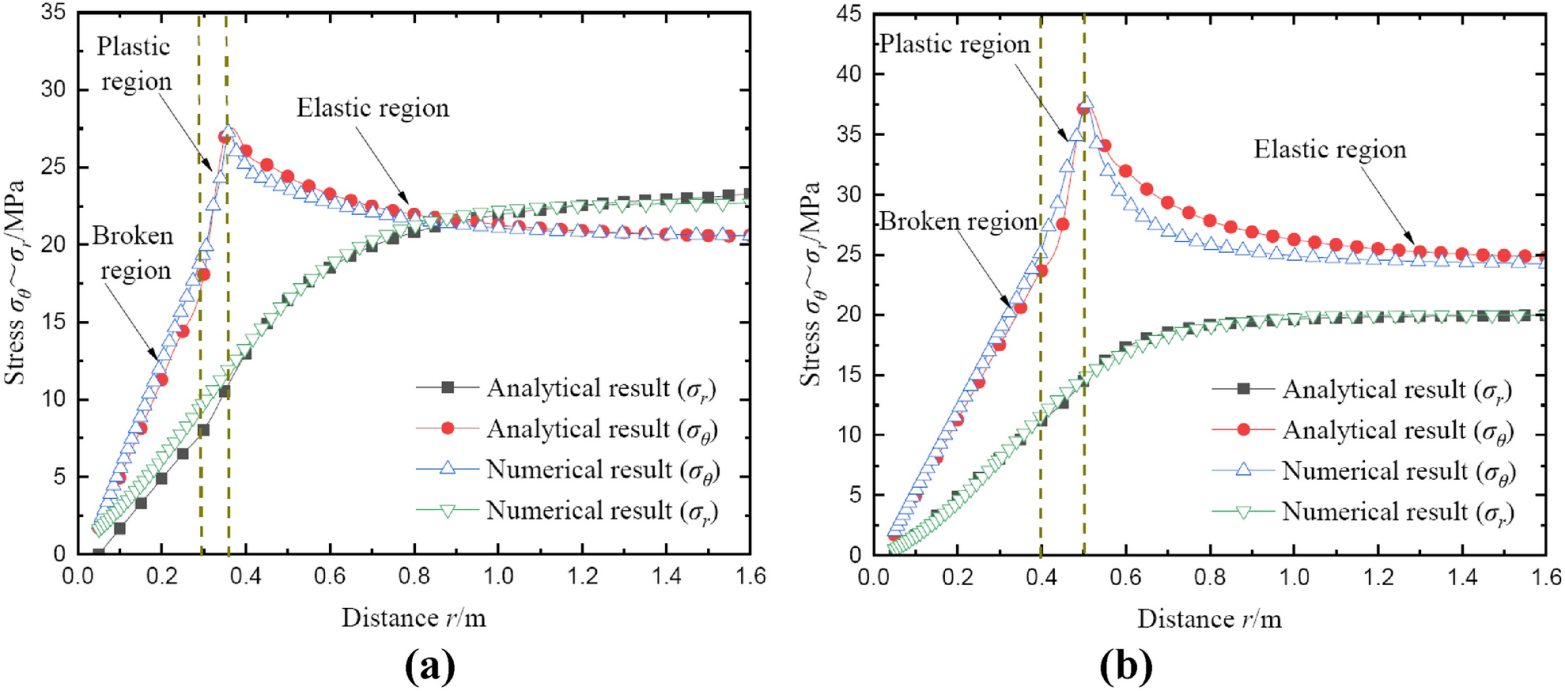
Comparison of stress results between analytical and numerical methods. (**a**) result of *θ* = 0°; (**b**) result of *θ* = 90°.

Figure 19 shows the comparison of stress results between analytical and numerical methods at polar angle *θ* = 0° and* θ* = 90°. Firstly, the results indicate that the radial and tangential stresses obtained by the analytical method are in good agreement with the results of the finite element method. Secondly, for the tangential stresses, the maximum error of the analytical method is 10.56% compared with the FEM. For the radial stress, the maximum error is only 6.94%. These results demonstrate that the analytical method proposed in this study is reasonable and effective.

**Fig. 20:**
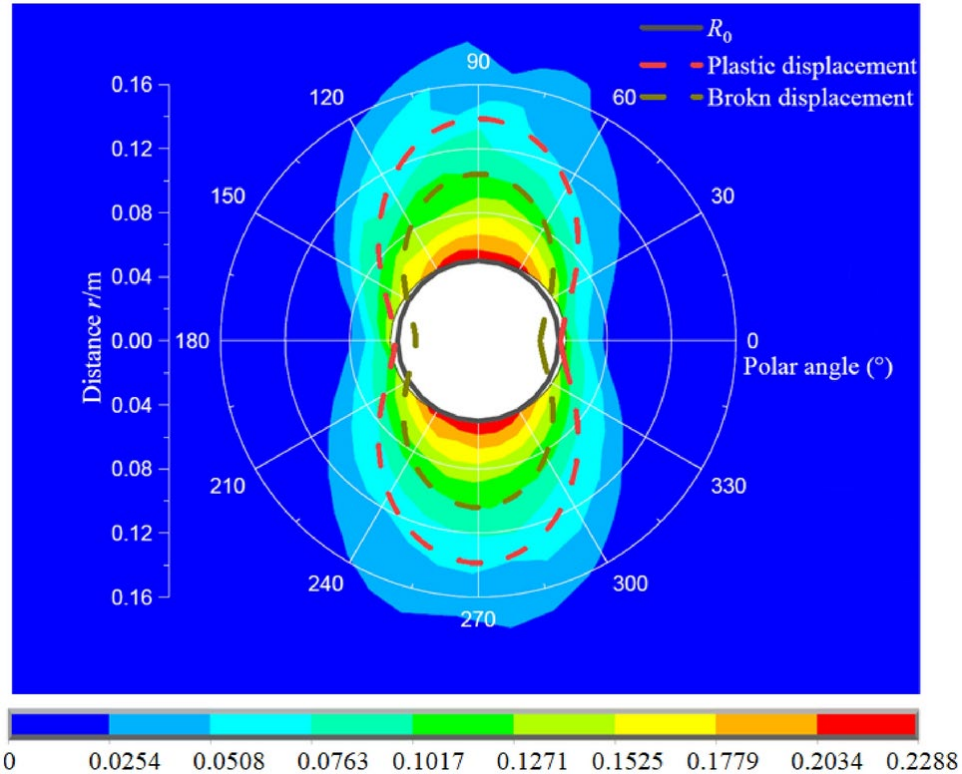
Comparison deformation distribution between analytical and numerical methods (Unit: m).

Figure 20 presents the comparison of displacement results between analytical and numerical methods displacement, it can be seen that the displacement distribution shape is similar, but distribution range of numerical simulation is slightly greater than that in analytical results. Two methods all indicates the top–bottom of borehole have already occurred serious damage, which is closely related to the lateral pressure coefficient. The major reason for the difference is the essence of the calculated methods. The analytical method relies on strict geometric model and boundary conditions, but a deformation body is adopted in the numerical method. Therefore, combing Fig. 19 and 20, the numerical results whether in stress or displacement are well consistence with the analytical results, which also validates the correctness of the present method.

## Discussion

This paper provides an analytical method for determining the broken, plastic softening, and elastic region for coal around borehole, which depends on the elastic–plastic theory. However, there are also some questions to be explored and researched in detail.

The plastic softening parameters selection of coal is the first concern in the analytical calculation. The cohesion is an accepted parameter, but the internal friction angle is controversial. Some scholars believe that the attenuation of residual internal friction angle in the plastic softening stage is very small for coal, and then it can be ignored, while others hold that the internal friction angle is an effective parameter.^16–18,26–30,37,38^ The results of calculation in this study show that the radii of two regions all decrease nonlinearly with increases of residual internal friction angle, and the amplitude of attenuation is very serious (Figs. 15 and 16). Therefore, it cannot be considered mechanically that the residual internal friction is useful or should be ignored. It should be focused on the triaxial test data to determine its importance. For now though, this similar research is rare in published reports. For different rocks, there is a significant difference in their mechanical properties due to their mineralogical composition and structure, thus it is very hard to express its plastic softening characteristic by a general mathematic model. Based on the experimental data, establishing a modified model of plastic softening is essential for special coal, and it can effectively analyze the deformation distribution around borehole, which will be the key points for future study.

Besides, the intermediate principal stress coefficient is also an important parameter for the deformation distribution of coal around borehole. Although many researchers analyzed the deformation of surrounding rock based on Mohr–Coulomb and Hoek–Brown criterion, but they ignored the influence of intermediate principal stress. Then, the results are not following reality^18,26–28^ In fact, three principal stresses of coal are different due to its microstructure, especially in deep engineering. The experimental results also show that the strength limit of rock can increase by approximately 30% considering the effects of intermediate principal stress^31^ Hence, using the Drucker-Prager criterion can solve this problem well. For deep rock, there is a broken region after excavation. Thus, how to divide the deformation regions of coal around borehole is very important in the analytical model. Three-stage constitutive model used in this study is more realistic for real working conditions. From the calculated results, it can be seen that the three regions do not change monotonically with the intermediate principal stress coefficient, and there has been hardly any research on this characteristic in the past (Fig. 12). To better assess the deformation mechanisms of coal around borehole, it is of great significance to make a profound study of the influence of intermediate principal stress on deformation distribution by employing different strength criteria, and it will be a great point of exploration.

It should be noted that the analytical method proposed in this study can be used in homogeneous coal or relatively simple coal seam. For the complex working conditions, such as high gas content and high temperatures also needs further verification. Because the constitutive relationship and softening model of coal will change under complex conditions, which affects the accuracy of the results.

## Conclusions

The deformation and failure of coal around borehole under different stress conditions are researched based on an analytical method proposed in the present study, and the influences of different parameters on stress and deformation distribution are also investigated in detail. The rationality of the analytical method is verified by comparison with the results of FEM. To sum up, the main conclusions are obtained as follows:

1. The constitutive model is the first important issue, and a simplified constitutive model is conducted out according to the deformation states in different depths of coal during the mining process instead of the full stress–strain model of coal. Then, based on three-stage constitutive model and DP strength criterion, an analytical solution of coal around borehole under different stress conditions including stress and deformation of the broken, plastic softening, and elastic region is deduced. Meanwhile, the plastic softening and dilatancy of coal are also implanted into the analytical solution.
2. The lateral pressure coefficient is a key factor, that predominantly affects the deformation shape of coal around borehole, and other parameters have almost no influence on the deformation shape. A large lateral pressure coefficient corresponds to a small radius of the broken and plastic softening regions. The radii of two regions increase significantly with an increase in vertical stress, but decrease clearly with an increase in initial cohesion and internal friction angle. Similarly, the larger the borehole radius, the greater the deformation ranges. The radii of two regions are not monotonous with the intermediate principal stress coefficient, there is almost an inflection point for the intermediate principal stress coefficient corresponding to the minimum radii of two regions, and the value is approximately *b* = 0.7.
3. The plastic strain softening characteristics of coal around borehole can be reflected by residual cohesion and internal friction angle. The smaller the residual value, the more severe the deterioration of coal performance and even leading to greater deformation ranges. However, for the residual internal friction angle, it should be focused on the triaxial test data to determine its importance. It’s worth noting that the influences of residual strength parameters are different from other parameters, which can be increased according to grouting during construction to improve the safety and stability of coal around borehole. Additionally, the borehole diameter should be reduced as possible under the same conditions to avoid the effects of drilling disturbance.

## Data Availability

The data used to support the findings of this study are available from the corresponding authors upon request.
